# Galyean Appreciation Club Review: revisiting nutrition and health of newly received cattle—what have we learned in the last 15 years?

**DOI:** 10.1093/jas/skac067

**Published:** 2022-03-05

**Authors:** Michael L Galyean, Glenn C Duff, J Daniel Rivera

**Affiliations:** 1 Department of Veterinary Sciences, Texas Tech University, Lubbock, TX 79409, USA; 2 Clayton Livestock Research Center, New Mexico State University, Clayton, NM 88415, USA; 3 Southwest Research & Extension Center, University of Arkansas, Hope, AR 71801, USA

**Keywords:** bovine respiratory disease, cattle, diagnosis, management, nutrition

## Abstract

Our objective was to review the literature related to the health and management of newly received cattle published since a previous review by Duff and Galyean (2007). Bovine respiratory disease (**BRD**) continues to be a major challenge for the beef industry. Depending on disease severity, animals treated for BRD have decreased performance and lowered carcass value. Diagnosis of BRD is less effective than desired, and progress on developing real-time, chute-side methods to diagnose BRD has been limited. Systems that combine lung auscultation with temperature and body weight data show promise. Assessment of blood metabolites and behavior monitoring offer potential for early identification of morbid animals. Vaccination and metaphylaxis continue to be important tools for the prevention and control of BRD, but antimicrobial resistance is a concern with antibiotic use. Dietary energy concentration and roughage source and level continue to be important topics. Mineral supplementation has received considerable attention, particularly the use of organic vs. inorganic sources and injectable minerals or drenches given on arrival. The use of probiotics and prebiotics for newly received cattle has shown variable results, but further research is warranted. The health and nutrition of newly received cattle will continue to be an important research area in the years to come.

## Lay Summary

Bovine respiratory disease (**BRD**) is a significant economic and animal welfare challenge for the beef industry. Experiments related to the health and management of newly received cattle published in the last 15 yr were reviewed. Limited progress is being made in developing accurate, real-time methods for diagnosis of BRD, and overall, diagnosis is less effective than desired. Measurement of lung and heart sounds combined with rectal temperature have been studied as diagnostic tools, as well as measurement of blood metabolites and remote monitoring of behavior. Vaccination for viral and bacterial BRD agents and mass treatment of cattle with antibiotics continue to be important tools for prevention and control of BRD, but the development of antimicrobial resistance is a concern. Energy and roughage concentration as well as roughage source continue to be important dietary considerations, as does mineral supplementation, with mineral source and injectable minerals receiving significant research attention. Probiotics and prebiotics fed to newly received cattle have shown variable results in terms of effects on the incidence of BRD and animal performance. Additional research is needed to define optimal diagnostic, management, and nutritional practices for newly received cattle.

## Teaser

Management and nutritional research conducted over the past 15 yr related to the health and nutrition of newly received beef cattle is reviewed.

## Introduction

Issues associated with the health and management of newly received cattle continue to pose significant animal welfare and economic challenges for the beef industry. The National Animal Health Monitoring System survey data (NAHMS, 2013) indicated that 16.2% of all cattle placed in feedlots were affected by bovine respiratory disease (**BRD**), which was the most common cause of morbidity and mortality in feedlots. Considering cow-calf, backgrounding, and stocker segments of the industry, the percentage of cattle with BRD is likely much greater than the NAHMS (2013) estimate for feedlots. Stresses associated with weaning, marketing, transportation, commingling of cattle from various sources, adaptation to new surroundings, and introduction of novel feed ingredients and feeding systems often lead to decreased intake by newly received cattle, further exacerbating immune and physiological challenges associated with BRD. Affected cattle are treated with injectable and feed-grade antibiotics. Economic losses resulting from mortality decreased performance, and diminished carcass value likely exceeds US$2 billion per year in the United States (Wilson et al., 2017a). Mortality is a direct loss, but the magnitude of performance and carcass losses depend on the severity of the disease (Holland et al., 2010; Brooks et al., 2011; Reinhardt et al., 2012; Wilson et al., 2017b; Blakebrough-Hall et al., 2020b), with greater losses associated with an increased number of treatments and severity of lung lesions. Segregating cattle with multiple BRD treatments and feeding to an acceptable carcass endpoint could provide an opportunity to increase the value of treated animals (Holland et al., 2010). Although often assumed to be a disease associated with highly stressed cattle sourced through auction markets, BRD occurs throughout the feeding period, with the incidence being later for high-performing cattle than for high-risk cattle (Theurer et al., 2021).

Approximately 15 yr ago, Duff and Galyean (2007) reviewed the literature related to advances in the nutrition and management of newly received beef cattle, with a focus on highly stressed and thereby high-risk cattle. In this inaugural review supported by the Galyean Appreciation Club, we reprise that previous effort and provide an update on advances in this important area that have been reported in the literature in the last decade and a half.

### Defining and assessing health of newly received cattle

#### Diagnosis—sensitivity and specificity

Accurate diagnosis of BRD continues to be a challenge. In the context of tools for diagnosis of disease conditions, sensitivity is the ability of the test to correctly classify the disease condition (i.e., true positive rate), and specificity is the ability of the test to correctly classify the absence of the disease condition (i.e., true negative rate). White and Renter (2009) suggested there is no “gold standard” for diagnosing BRD, noting that clinical illness or lung lesions were relatively poor at correctly classifying truly diseased cattle; however, lung lesions assessed at slaughter were more accurate than clinical illness signs for BRD diagnosis. Although evaluation of lung lesions is an important research tool, it has no clinical value in real-time diagnosis of BRD.

To evaluate the economic consequences of subclinical BRD, Blakebrough-Hall et al. (2020b) examined four BRD diagnosis methods including the number of BRD treatments, pleural lesions at slaughter, lung lesions at slaughter, and clinical BRD status defined using both treatment records and lung and pleural lesions. Economic returns decreased with the severity of BRD, and subclinical BRD decreased returns compared with healthy animals. Cattle treated for BRD can reach similar compositional endpoints (e.g., 12th rib ultrasound fat thickness) to untreated cohorts; however, carcass yield and quality grade may be less desirable (Wilson et al., 2017b).

Visual diagnosis of BRD typically includes nasal or ocular discharge, lethargy, emaciated body condition, labored breathing, or any combination, and evaluation of rectal temperature (>37.5 °C; Duff and Galyean, 2007). Nichols (2014) described the “DART” method (depression, appetite, respiration, and temperature) to score cattle for BRD treatment. Signs of BRD in the DART system include a depressed appearance, droopy head or ears, weakness or “knuckling” of hind fetlocks, lack of appetite and a gaunt appearance, isolation, labored breathing, coughing, nasal discharge, eye discharge, and weakness, with scores scaled from 0 (no symptoms) to 4 (very severe exhibition of several symptoms). Rectal temperature is measured for scores of 1 or 2, with antibiotic treatment given only when temperatures are ≥40 °C. Cattle given a score of 3 or 4 are treated regardless of temperature (Nichols, 2014). Although rectal temperature is easy to obtain and inexpensive, operator variability can be a problem (Bewley et al., 2008), as well as effects of the environment, handling and movement, hide color, time of day, etc. Thus, some flexibility might be desirable in setting cutoff points for BRD treatment, but research is needed in newly received cattle to better define the options. Personnel need to be aware that morbid animals may mask their vulnerability (Weary et al., 2009); thus, training to correctly identify morbid animals will continue to be important for feedlot managers.

#### Diagnostic techniques and tools

Diagnosis of the clinical signs of BRD is marginal at best, and diagnostic tools are needed for accurate evaluation, but few practical alternatives have been identified. Richeson et al. (2018) summarized three methodologies for characterizing cattle behavior: three-axis accelerometers; systems for evaluating feeding and watering behavior; and triangulation or global positioning for cattle location and movement in pens or on pasture. The behavior monitoring systems detected BRD early, with variable timing relative to standard clinical BRD diagnosis. Nonetheless, we need to better understand their cost–benefit ratio because treatment costs could increase as a result of improved sensitivity compared with traditional diagnostic methods. Commercial success and adoption will depend on improved diagnosis, decreased labor costs, decreased mortality, and increased performance that can offset the cost of the system, as well as the ability to integrate these systems within current management systems (Richeson, 2020).

Low feed intake by newly received cattle has long been considered a factor associated with BRD; thus, feeding behavior could be an early predictor of health in newly received cattle. Wolfger et al. (2015) reported that the mean intake per meal, mean mealtime, and frequency of meals could predict BRD in feedlot cattle 7 d before visual diagnosis of symptoms. Commercial application of feeding behavior to detect BRD will require development of predictive algorithms and testing under multiple management scenarios.

Blood metabolites have often been considered as potential diagnostic tools. Oosthuysen et al. (2016) reported that the percentage of hemoglobin saturated with O_2_ was negatively but lowly correlated with mortality (−0.08) and might be a possible tool for early detection of BRD. In addition, blood pH and glucose were correlated with first and second medical treatments, and blood lactate was correlated with first medical treatment and mortality. Montgomery et al. (2009) reported that plasma glucose and lactate concentration decreased linearly with the number of times newly received heifers were treated for BRD. Blakebrough-Hall et al. (2020a) used ^1^H NMR metabolomics to search for biomarkers of BRD. Phenylalanine, lactate, hydroxybutyrate, tyrosine, citrate, and leucine were identified as important metabolites in calves that were morbid from BRD. ^1^H NMR metabolomics could have potential as a tool in diagnosing BRD, but as with all blood measurements, the approach is far from a chute-side technique and will require homing in on metabolites and rapid testing methods to be of practical utility.

As noted previously, the febrile response has traditionally been a key element of BRD diagnosis, and recent studies have evaluated combining rectal temperature data with other metrics. Nickell et al. (2021) evaluated the Whisper On Arrival system, a patented chute-side technology developed to predict the risk of BRD in individual cattle at receiving. The technology uses a sound-collection device to measure heart and lung data, which are combined with rectal temperature and body weight (**BW**) data in a proprietary algorithm to determine a “Treat” or “Do Not Treat” recommendation. Thus, the system compares the likelihood of BRD at arrival, which differs from the original Whisper Veterinary System Stethoscope that was used as a supplement to traditional BRD diagnosis (Nickell et al., 2020). Across four study sites, using the Whisper On Arrival technology decreased antibiotic use by 11% to 43% (Nickell et al., 2021) compared with metaphylaxis, but applying the system results in additional processing costs associated with labor, time, and equipment. Future research in this area should compare system-based treatment recommendations vs. randomly treating a similar proportion of the population.

### Management factors affecting BRD

#### Preconditioning

Preconditioned cattle gain faster and require fewer antibiotic treatments during the receiving period (Richeson et al., 2012). Despite their value, preconditioning programs have been slow to be widely adopted by the beef cattle industry. Preconditioning programs typically include a vaccination protocol, a 45-d weaning period, dehorning and castration, adaptation to feed bunks and water troughs, and individual identification (Wilson et al., 2017a). Several states have implemented and promoted preconditioning programs, which have been developed in anticipation of premiums for preconditioned calves vs. non-preconditioned contemporaries but the return on investment has long been a concern of producers. Thrift and Thrift (2011) reported that buyers paid premiums ranging from US$1.43 to US$6.14 per 45.4 kg, but increased profit was not always realized (−US$89.92 to US$53.71 per calf). Facilities are required to house the animals during the preconditioning period, which could be a challenge for some cow-calf operations, and loss in BW (shrink) and potential death loss before sale is borne by the producer. Despite challenges, cow-calf producers could realize a premium through a reputation for integrity or by marketing their animals through special preconditioning sales (Thrift and Thrift, 2011).

The time preconditioned cattle are held after weaning has varied and depends on individual producer circumstances, facilities, feed costs, and marketing strategies. Anderson et al. (2016) reported that 21 d could be more beneficial to the cow-calf producer than 42 d by decreasing feed costs without negatively affecting performance and carcass characteristics.

For producers who opt to precondition, Boyles et al. (2007) suggested that fence-line pasture weaning (calves have contact with their dams) is an acceptable method. The incidence of BRD was only 15% for pasture-weaned calves, double for truck-weaned calves, and 2.5 times greater for calves weaned in a drylot. Acclimating pasture-weaned calves to feed bunks did not improve health or performance (Bailey et al., 2016), so a low-input program could be a means of decreasing the costs of preconditioning.

Commingling cattle is another source of stress during feedlot receiving and possibly during preconditioning; however, commingling heifers from two to four different sources did not affect performance or BRD during a 56-d receiving period (Wiegand et al., 2020). The use of small groups might have contributed to the lack of negative results with commingled heifers. Commingled preconditioned and non-preconditioned steers on winter wheat pasture had a greater abundance of nasal *Mannheimia* than non-commingled preconditioned or non-preconditioned steers (Brooks et al., 2021). Further research is needed on how management factors affect the timing of colonization of the nasopharynx with *Mannheimia haemolytica* and how colonization relates to BRD.

Late-castrated bulls had poorer performance and greater morbidity than early castrated bulls (Massey et al., 2011). With current marketing practices, intact bulls are often received in a feedlot or pasture setting. Purchasing bulls over steers will decrease performance during stocker receiving phases, and more newly castrated bulls will be treated for BRD than steers, thereby increasing medical and labor costs (Ratcliff et al., 2014).

Based on the current literature, we highly recommend that cattle be preconditioned before shipment to the backgrounding or growing facility. Nonetheless, additional research to define the key elements of preconditioning programs that can be applied by producers in a cost-effective manner might aid industry adoption.

#### Transportation

Beef cattle can be transported as many as six times in their lifetime (Schwartzkopf-Genswein et al., 2016), including transport from farm or ranch to auction facility, auction facility to order buyer facility, order buyer facility to stocker or grower facility (pasture or drylot), stocker or grower facility to feedlot, and finally to a packing plant. Factors affecting welfare during transportation include loading density, transportation duration, trailer design and ventilation, driving, handling quality, road and environmental conditions, and fitness of the cattle (Schwartzkopf-Genswein et al., 2016). A beef quality assurance program is available (https://www.bqa.org/programs/transportation-program) that promotes proper handling and transportation of cattle to potentially decrease sickness in newly received calves and carcass defects in finished cattle.

Driver experience and skill can affect the amount of shrink animals experience during transportation. Cattle transported by drivers with ≥6 yr driving experience had less shrink than those transported by drivers with ≤5 yr experience (Gonzalez et al., 2012), suggesting that drivers with greater experience are more conscientious about starting, stopping, turning, and may have better animal handling skills. Truck compartment could be important, as Wahrmund et al. (2012) reported that heifers transported in the bottom deck nose and upper deck rear sections of a trailer had increased BRD compared with cattle in other compartments.

Shrink (loss of BW from gut fill and loss of body fluid and tissue) is a common marker of stress during transportation. Multivariable regression analysis indicated that variables associated with BRD morbidity included shrink, gender, the season of arrival, cohort size, mean arrival BW, arrival year, and two-way interactions between shrink and arrival BW, gender, and season (Cernicchiaro et al., 2012). In the United States, cattle can be transported for 28 h, and in Canada, the maximum transport duration is 52 h (Schwartzkopf-Genswein et al., 2016); thus, with long-haul cattle, it may be advantageous to implement rest stops. Cooke et al. (2013b) reported that including 2-h rest stops during a 1,290-km transport prevented increased nonesterified fatty acid and cortisol concentrations compared with transported cattle that did not receive rest stops. Despite the changes in these markers of stress, receiving period performance was not altered by rest stops. Feed and water deprivation during transport are the likely causes of changes in stress markers (Marques et al., 2012).

#### Vaccination

In general, BRD is a multifaceted disease that can result from viral pathogens weakening the immune system and physically damaging the epithelial mucosa of the upper respiratory tract, which can allow bacterial pathogens to proliferate and limit the ability of the animal to ward off the insult. Stresses associated with marketing, transportation, and receiving exacerbate the challenges, and animals can ultimately succumb to bacterial pneumonia. Thus, proper vaccination against viral agents associated with BRD is an important management practice.

Vaccinating calves against respiratory pathogens before feedlot entry is a valid strategy for improving cattle health and performance during receiving (Lippolis et al., 2016). Vaccination against BRD 15 d before weaning plus revaccination 15 d before feedlot entry decreased the incidence of BRD in feedlot cattle (Schumacher et al., 2019) compared with vaccination on feedlot arrival. Poe et al. (2013) reported that there were no benefits or detriments to performance or morbidity rates from delaying BRD vaccination by 14 d, and Richeson et al. (2008) reported that delaying vaccination for 14 d increased average daily gain (**ADG**) compared with a modified live virus vaccination on arrival. Sharon et al. (2013) reported that vaginal temperature was increased for 1 to 3 d in heifers after vaccination with a modified-live viral vaccine, with a more pronounced increase in heifers that were vaccinated 14 d after arrival vs. on arrival. These results suggest that producers should consider febrile responses induced by vaccination when they develop BRD treatment protocols.

Duff and Galyean (2007) suggested the need for further evaluation of the effects of lysine on bovine herpes virus 1 (**BHV-1**). As a follow-up to this suggestion, Sharon et al. (2014) evaluated the effect of supplemental lysine on serum infectious bovine rhinotracheitis (caused by BHV-1) titer in response to intranasal or intramuscular respiratory-virus vaccination in neonatal calves. Supplemental lysine affected nitrogen metabolism, but it did not alter the response to infectious rhinotracheitis vaccination.

Readers are referred to Richeson and Falkner (2020) for an excellent review on the use and timing of vaccines in beef cattle. Respiratory vaccines are a relatively inexpensive tool to decrease the occurrence of BRD, resulting in improved animal welfare and performance, and we recommend that producers work with veterinary professionals on specific vaccine recommendations.

#### Metaphylaxis programs

Injectable antibiotic therapy given under veterinary guidance continues to be the primary means of treating cattle with BRD, with NAHMS (2013) indicating that virtually all cattle diagnosed with BRD were given an injectable antibiotic. Metaphylaxis is an important management tool, with NAHMS (2013) noting that 59.3% of feedlots used metaphylaxis for some cattle, with the focus on cattle weighing less than 317 kg. From an economic perspective, Dennis et al. (2018) estimated that the cattle feeding industry receives a net return value of US$532 to US$680 million per year from using metaphylaxis programs.

Duff and Galyean (2007) focused their review on metaphylaxis programs that used oxytetracycline, tilmicosin, and florfenicol, but it was noted that tulathromycin, which was approved for use in beef cattle in 2005, showed promise in initial studies. Since the 2007 review, additional antibiotics have been approved and evaluated in research studies.

Tilmicosin phosphate continues to be widely used by the beef industry as a metaphylaxis treatment. Reuter et al. (2008) evaluated the response to a lipopolysaccharide injection in 247-kg steers fed different sources and levels of dietary energy with or without injection of tilmicosin. A roughage-based diet and a higher concentrate diet were fed to equalize energy intake to the roughage diet increased serum concentrations of cytokines compared with a higher concentrate diet fed ad libitum. Tilmicosin also increased cytokines, but only in the roughage and restricted higher concentrate diets, indicating that tilmicosin might have direct immunomodulatory effects that are independent of its antimicrobial actions.

Since 2007, tilmicosin has been compared with several other antibiotics for use in metaphylaxis programs. Word et al. (2020) reported that the percentage of calves treated for BRD decreased from 76.7 in control calves to 46.4 and 56.5 with tilmicosin and ceftiofur, respectively. Despite mass treatment on arrival, total antimicrobial use did not differ among treatments, reflecting greater treatment success rates in the two metaphylaxis groups. Van Donkersgoed and Merrill (2013) compared tilmicosin and tildipirosin as metaphylaxis treatments in 336-kg crossbred steer calves at a commercial feedlot. Tildipirosin-treated calves had a lower percentage of initial treatments for BRD than tilmicosin-treated calves (16% vs. 30%), but relapse rates and mortality were not affected by treatment. Despite the difference in morbidity, tilmicosin had a net economic advantage over tildipirosin, reflecting its lower cost under the conditions of the study.

Word et al. (2021) compared metaphylaxis with tilmicosin and tildipirosin with a non-medicated control group in Mexican steers (213 kg BW) received at a commercial feedlot. Calves treated on arrival with tilmicosin (4.24%) and tildipirosin (2.06%) had a lower percentage of BRD than control calves (10.98%) during the initial 60 d of the experiment, with tildipirosin differing (*P* = 0.03) from tilmicosin. Performance and carcass characteristics for the overall feeding period were not affected by metaphylaxis treatments, and days on feed did not interact with metaphylaxis.

After it was approved for use in feed, Rivera et al. (2018) compared feeding tilmicosin (12.5 mg/kg of BW) for 14 d vs. a non-medicated control diet in two 56-d experiments with heifers. In the first experiment, heifers (196 kg) received metaphylaxis before shipment, and dietary treatment with tilmicosin was initiated when 10% of the heifers were diagnosed with BRD. Feeding tilmicosin decreased performance for the first 28 d of the experiment but had no effect on BRD morbidity. In the second experiment, heifers (227 kg) did not receive metaphylaxis before shipping, and because of a high morbidity rate at initial processing, feeding tilmicosin commenced the day after arrival. Treatments did not affect performance or morbidity, with an average of 87.3% of the heifers treated for BRD, and total medication cost per animal was increased by feeding tilmicosin.

Tulathromycin is an effective therapeutic antibiotic (approximately 80% first-treatment success rate for both tulathromycin and tildipirosin; Theurer et al., 2018), and the results of several studies have indicated that it also is highly effective in metaphylaxis programs. Tennant et al. (2014) compared arrival metaphylaxis with tilmicosin or tulathromycin with a non-treated control group in 2,336 beef steers (312 kg) at a commercial feedlot. Tulathromycin (2.1%) and tilmicosin (5.7%) decreased BRD morbidity vs. the control group (14.3%), with a significant difference between the two antibiotics, and financial returns were increased by metaphylaxis treatments. Surprisingly, lung lesions recorded at slaughter were noted in 64.3% of the cattle, with no differences among treatments. Ball et al. (2019) compared arrival treatment with tulathromycin and tilmicosin in 232-kg steers and bulls sourced from auction barns. Treatments did not differ in BW and ADG throughout the 42-d study, but the percentage of calves treated once (28.4 vs. 53.4) or twice (4.6 vs. 28.4) for BRD was less with tulathromycin than with tilmicosin. The interaction of metphylaxis with tulathromycin and vaccination with a pentavalent modified-live virus respiratory vaccine was evaluated by Munoz et al. (2020) using 478 bull and steer calves (234 kg) sourced from auction markets. The metaphylaxis × vaccination interaction was not significant for BRD morbidity or performance variables. Tulathromycin decreased BRD treatments from 51.2% to 18.5% and increased performance compared with control, but vaccination had no effects on the response variables.

Several meta-analyses of literature data have been conducted to assess the efficacy and economic significance of metaphylaxis programs. Readers are referred to Nautrup et al. (2013), Abell et al. (2017), and O’Connor et al. (2019) for details on the findings of these useful reports, which provide summaries of outcomes with various antibiotics used in metaphylaxis programs.

Duff and Galyean (2007) summarized available data on antimicrobial resistance (**AMR**), which continues to be a major concern associated with the use of therapeutic antibiotics in metaphylaxis programs. Readers are referred to the excellent review by Cameron and McAllister (2016) regarding antimicrobial use and resistance in beef production systems. Concerns noted in this review included the role that pathogens resistant to antimicrobials could play in affecting the efficacy of antibiotic treatment regimens for infectious diseases in cattle and the potential effect of AMR associated with pathogens that originate in cattle and subsequently cause human illness (e.g., *Escherichia coli* and *Salmonella*). Thus, the role of BRD metaphylaxis in the propagation of AMR continues to be an important area of experimentation.

Checkley et al. (2010) evaluated the resistance of fecal generic *E. coli* isolates to seven antimicrobials in 288 calves (256 to 353 kg) sourced from auction barns in Canada. Treatments were control (no arrival medication), oxytetracycline in the diet for 14 d, and arrival medication with an injection of long-acting oxytetracycline. Approximately 81% of the calves showed no resistance on arrival, but the proportion of calves with resistant isolates increased for the two antibiotic treatment groups vs. control in the first 15 d after arrival. Across treatments, the odds of cattle having tetracycline-resistant isolates 24 h before slaughter were 6.4 times greater than at arrival, indicating that resistant isolates generally increased with days on feed.

Coetzee et al. (2019) used data from bovine samples submitted to the Iowa State Veterinary Diagnostic Laboratory to assess AMR in isolates of *M. haemolytica*, *Pasteurella multocida*, and *Histophilus somni*. An increased number of antibiotic treatments per animal was associated with a greater chance of isolates being resistant to at least one commonly used antibiotic. Moreover, initial treatment with a bacteriostatic antibiotic (e.g., tulathromycin) followed by treatment with a bactericidal antibiotic (e.g., ceftiofur) yielded the highest probability of resistant *M. haemolytica* isolates.

In 169 bull and steer calves (229 kg) sourced from auction markets and treated with tulathromycin on arrival, 16% had *M. haemolytica* isolated from nasopharyngeal swabs on arrival, with 3.7% showing multidrug resistance (Snyder et al., 2017). Ten to 14 d after arrival, *M. haemolytica* was detected in 72.8% of the calves, with 99.2% of these isolates showing multidrug resistance. Because a non-mediated control group was not included in the experiment, the authors concluded further research was needed to understand the role of metaphylaxis in multidrug resistance in *M. haemolytica*.

Doster et al. (2018) sampled feces from 15 steers (300 to 400 kg BW) that were not exposed to antibiotics or mass-treated with tulathromycin on the day of treatment and 11 d later. Sequencing of isolated DNA was conducted to identify antimicrobial genes. The resistome and microbiome did not differ between treatment groups but changed between sampling times, with a greater antimicrobial relative abundance for the tetracycline and macrolide-lincosamide-streptogramin classes on day 11 vs. day 1. Holman et al. (2019) used 18 steers and 18 heifers (301 kg BW) to compare control, injection with long-acting oxytetracycline, and injection with tulathromycin on the nasopharyngeal and fecal microbiomes. Samples were collected on-farm before shipment to the feedlot, at the time of initial processing and application of treatments, and 2, 5, 12, 19, and 34 d later. Both the nasopharyngeal and fecal microbiomes were altered over time, with the effects of the two antibiotic treatments being greatest on days 2 and 5. Antibiotic treatments increased antibiotic resistance genes relative to control.

Overall, current data suggest the need for continued concern regarding the effects of antibiotic use on AMR in the beef industry. As therapeutic treatment and metaphylaxis for BRD represent significant components of overall antibiotic use, additional controlled studies are needed to assess the longitudinal changes in AMR as cattle progress through  the feeding period, with a particular focus on understanding the  effects of therapeutic treatment vs. metaphylaxis.

#### Anti-inflammatory treatment

Cooke (2017) reviewed the significance of stress-related inflammation in newly received cattle. Stressors associated with weaning, transport, and management of newly received cattle increase plasma concentrations of acute-phase proteins, which seems correlated with animal performance (Cooke, 2017). One approach to manage this inflammatory response would be the use of nonsteroidal anti-inflammatory drugs in newly received cattle. For example, Cooke et al. (2013a) used 45 beef steers (228 kg) to assess the effects of saline vs. flunixin meglumine injection at loading and unloading for a 1,280-km transport followed by a 28-d receiving period. A non-transported control group that received saline injections to match loading and unloading times also was included. In transported calves, administration of flunixin meglumine decreased the acute-phase proteins haptoglobin (days 1 and 4) and ceruloplasmin (days 4 and 7) after arrival relative to transported calves that received saline only but receiving period performance did not differ between transportation treatments.

Guarnieri Filho et al. (2014) used 84 steers (252 kg BW) transported 1,440 km to evaluate the effects of meloxicam (1 mg/kg BW) vs. a lactose monohydrate control and a non-transported control group. Treatments were applied at loading and unloading by oral drench and in the feed for day 2 through day 7 of a 21-d receiving period. Meloxicam increased dry matter intake (**DMI**) for the first 7 d after transport but not for the overall 21-d period, but ADG and G:F were greater for the 21-d period for meloxicam-treated vs. transported control steers. Plasma haptoglobin was increased in transported control steers vs. meloxicam and non-transported control steers on day 5 of the receiving period, and plasma ceruloplasmin was less in meloxicam vs. transported control steers on days 5 and 7. Van Engen et al. (2019) compared pre-transport and arrival treatment with meloxicam (oral bolus of 255 mg) vs. control in 168 auction-sourced beef steers (251 kg BW). In contrast to the results of Guarnieri Filho et al. (2014), performance, BRD morbidity, and serum or plasma concentrations of several biomarkers of stress or inflammation did not differ among treatments for the 42-d receiving period nor was performance affected during a subsequent feedlot finishing period.

Based on limited extant data, results regarding the value of anti-inflammatory drugs in newly received cattle are mixed. Further research is needed to identify situations (e.g., type of and source of cattle and length of transportation) that would most likely benefit from the use of anti-inflammatory drugs and optimal approaches for use (e.g., dose and timing of administration).

### Nutritional and dietary factors

#### Energy

As noted by Duff and Galyean (2007), the effects of energy concentration in the diets of newly received cattle are challenging to assess because changing energy concentration is typically accomplished by changing dietary ingredient composition. The ingredient of choice to elicit changes is most often roughage, as is reflected in the early work of Lofgreen et al. (1981), in which a 75% concentrate receiving diet, with or without free-choice access to millet or alfalfa hays was compared with the hays alone. In summarizing studies conducted by Lofgreen and colleagues over a period of several years, Rivera et al. (2005) noted that increasing dietary roughage level decreased BRD morbidity slightly, but it also decreased ADG and DMI, resulting in an economic disadvantage for higher roughage levels. Readers are referred to Richeson et al. (2019), who recently provided an insightful review of the effects of energy and roughage levels on the health and performance of newly received cattle.

Obviously, changing dietary energy concentration by changing roughage level (or other ingredients) can result in numerous changes in dietary components (e.g., starch, protein, ether extract, minerals, and vitamins). High starch concentrations in receiving diets might induce acidosis in the gastrointestinal tract, with increased lipopolysaccharide in the rumen and markers of an inflammatory response in the serum (Gozho et al., 2005), perhaps resulting in conflating effects of acidosis with the diagnosis of BRD (Richeson et al., 2019). Nonetheless, diets with low vs. high starch content fed at two different energy concentrations (Berry et al., 2004) did not result in major differences in morbidity between starch levels or energy concentrations.

So how do we sort out the direct effects of energy intake per se on the health and performance of newly received cattle? One option is to feed diets that differ in dietary components, and thereby energy concentration, at the same energy intake. As noted previously, Reuter et al. (2008) used this approach to compare 30% vs. 70% concentrate diets fed at equal NEg intake on the inflammatory response in beef steers, but this method is not common in applied receiving studies. Recently, Spore et al. (2018, 2019) used 354 heifers (214 kg BW) purchased from auction markets to evaluate diets with NEg concentrations of 0.99, 1.10, 1.21, and 1.32 Mcal/kg in a 55-d study. Diets contained 40% wet corn gluten feed, with dry-rolled corn used to modify the energy concentration. The feeding level was decreased with increasing energy concentration (95%, 90%, and 85% of the 0.99 Mcal/kg diet for the 1.10, 1.21, and 1.32 Mcal/kg diets, respectively). Calves in all treatments received enrofloxacin at arrival processing, resulting in average morbidity of 12.2% that did not differ among treatments. Daily gain did not differ among treatments, but by design, DMI decreased and G:F increased with increasing energy concentration (Spore et al., 2019). Dietary energy concentration did not affect serum haptoglobin concentrations or antibody titers to bovine viral diarrhea type I or infectious bovine rhinotracheitis (Spore et al., 2018), and the authors concluded that programmed feeding of high-energy diets was a viable approach for newly received cattle. Calculations based on DMI and BW data presented by the authors indicate that after accounting for maintenance requirements, NEg intake above maintenance was 3.2%, 9.5%, and 18% greater for the 1.10, 1.21, and 1.32 Mcal/kg diets for the 55-d study, so unfortunately, the experimental approach did not fully equalize energy intake across the diets. Additional studies using this general approach but feeding to a specific intake of NEg rather than a relative percentage of the DMI would be useful. Moreover, evaluating the effects of different energy concentrations fed at a fixed NEg intake in calves that are not mass treated with an antibiotic would be useful in terms of assessing the health effects of changes in dietary energy concentration.

#### Protein

Not much new information has been reported in the past 15 yr with respect to the effects of protein on the health and management of newly received calves. Waggoner et al. (2009) reported that lipopolysaccharide infusion in 250-kg steers decreased plasma concentrations of most amino acids measured vs. saline infusion, but there was no interaction of lipopolysaccharide with dietary crude protein (CP) concentration (14.5% vs. approximately 16% CP) or source (degradable vs. undegradable) for plasma amino acid concentrations. Negative effects of lipopolysaccharide on N retention were mitigated by the higher CP diets regardless of source. Activation of the immune system and inflammatory response associated with stress and BRD in newly received calves would be expected to increase the need for amino acids. With low feed intake in newly received cattle and thereby a low supply of amino acids, this need for amino acids would be met via catabolism of muscle tissue (NASEM, 2016).

Given low feed intake, the extent to which an increased need for amino acids could be met by altering dietary protein concentration and source is open to question. Duff and Galyean (2007) noted that increasing CP concentration has been shown to increase ADG and DMI during receiving periods, although this has not always been the case (Lehmkuhler and Kerley, 2007). One might expect that greater performance would be associated with decreased BRD, but there is some evidence that BRD morbidity increases with increasing dietary CP concentration (Duff and Galyean, 2007). Increased BRD morbidity with higher protein might reflect a more intense inflammatory response, as evidenced by greater rectal temperature in calves challenged with bovine herpesvirus-1 and fed bermudagrass hay plus supplemental soybean meal vs. control calves fed bermudagrass hay only, possibly leading to a greater BRD treatment rate in protein-supplemented calves (Whitney et al., 2006). Given the lack of work in the past 15 yr, further research is needed on how dietary protein level and source affect BRD in newly received calves.

Protein deficiency negatively affects immune function (Li et al. 2007). Perhaps the protein status of newly received cattle before they are marketed, transported, and acclimated to a new environment is more important than the dietary protein concentration in receiving diets, as long as some reasonable minimum receiving diet CP concentration is provided. In most receiving studies, the cattle are sourced from auction markets, which results in a population with a greater risk for BRD and thereby a greater ability to test treatment effects. Nonetheless, the background—and thereby the nutritional status—of these calves is generally unknown to the researchers. Additional research focused on pre-shipment dietary modifications that could improve the protein status of calves and allow them to respond more effectively to an increased need for amino acids during the receiving period might prove worthwhile.

#### Roughage

Roughage is an important component in newly received cattle diets in terms of providing calves a familiar feed and potentially moderating BRD, but with the possibility of decreased performance associated with increased roughage (Rivera et al., 2005). Galyean and Hubbert (2014) noted that on an energy basis, roughages are expensive and often inconsistent in quality and composition; however, few studies have examined alternatives to traditional roughage (hay) sources in newly received cattle diets. Loya-Olguin et al. (2008) fed 67% concentrate diets with alfalfa hay that was ground or slice-baled (hay was chopped before baling) to 183 kg, high-risk steer calves. In theory, sliced bales maintain the integrity of the leaf and could result in less fines, which might improve quality vs. ground alfalfa. The authors concluded that sliced bales were more effective than ground alfalfa as a roughage source for newly received cattle, but additional research has not been reported to confirm these findings.

In addition to traditional roughage sources like hays, other highly digestible fiber sources have been examined for use in newly received cattle diets. Ponce et al. (2012b) compared a 65% concentrate receiving diet to two, wet corn gluten-based proprietary diets (Cargill Corn Milling, Blair, NE) in newly received beef heifers (average BW = 184 kg) during a 35-d receiving period. Greater ADG and DMI, as well as improved feed efficiency, were noted for cattle fed the 65% concentrate diet compared with the wet corn gluten-based diets; however, no differences were observed for BRD morbidity among treatments. Schneider et al. (2012) compared three diets in 261-kg crossbred steers purchased from livestock markets during 30- to 31-d receiving periods. Diets consisted of a control receiving diet with 35% alfalfa hay, 30% wet corn gluten feed, and 30% dry-rolled corn and two proprietary diets (RAMP and Test Starter) with a high concentration of wet corn gluten feed. The RAMP diet increased ADG compared with the control, but ADG by cattle fed Test Starter did not differ from the control. Morbidity was low at 8%, but the two proprietary diets had a higher incidence of morbidity. In a subsequent report, Schneider et al. (2013) compared the RAMP diet to a control diet over 2 yr with 259-kg steer calves. Receiving periods were 31 and 24 d in the 2 yr. The RAMP diet increased DMI in the first year but decreased it in the second year. The daily gain was increased in the first year but not the second, and BRD morbidity did not differ between treatments over 2 yr. Since these reports, RAMP diets have been widely adopted by the feedlot industry, indicating that digestible fiber sources are effective ingredients in receiving diets.

Byproduct sources of fiber besides corn byproducts have received limited attention. Woolsoncroft et al. (2018) compared two receiving diets that contained 30% roughage: one diet was a blend of soybean hulls (15% of dietary DM) and cottonseed hulls (15% of dietary DM), and the second diet consisted of only prairie hay at 30% of the dietary DM. Feeding prairie hay increased 56-d DMI compared with the hull combinations, but no differences in ADG or BW were noted, which led to an increase in gain efficiency for the hull-based receiving diet. No differences were observed in the first antibiotic treatment rate, but there was a tendency for fewer cattle-fed hay to require a second antibiotic treatment.

Using pelleted roughages could have advantages over traditional roughage sources, which can pose challenges with storage and handling; however, data are limited with regards to the use of pelleted feedstuffs in newly received cattle diets. Farran et al. (2001) fed a pelleted cottonseed hull and cottonseed meal (65:35) product included at 40% of the dietary DM compared with a receiving diet with alfalfa (40% of dietary DM) to 203-kg beef heifers for a 28-d receiving period. No differences were noted in ADG, morbidity, or mortality, but feeding the pelleted cottonseed hull and cottonseed meal increased DMI. Perhaps physically effective neutral detergent fiber (NDF) was greater for the alfalfa diet, thereby limiting DMI compared with the pelleted cottonseed hull and cottonseed meal diet. Peterson et al. (2015) fed a pelleted complete feed consisting of corn residue and grain byproducts and a standard receiving diet with 32% alfalfa to newly purchased beef cattle at two locations in Nebraska. Final BW, ADG, and feed efficiency were significantly greater, and DMI was less for the cattle fed the alfalfa diet compared with the pelleted complete feed. Cattle fed the control diet in one location had less second-pull morbidity than those fed the pelleted feed, whereas the opposite effect was noted at the second location. Unfortunately, the control diet in these studies contained monensin, but the pelleted feed did not have an ionophore, resulting in the potential for confounding. Current data are limited, but because hays can be expensive on a cost per nutrient basis and difficult to handle, alternative roughage sources warrant further investigation.

#### Silages

Duff and Galyean (2007) suggested that newly received cattle prefer dry hay to silage, and Preston (2007) felt that unfamiliarity with silage could potentially limit newly received cattle from reaching optimal DMI. Another concern with silage is that it could pose other risks for newly received cattle. Toxins associated with improper ensiling such as *Clostridium botulinum* and molds could affect DMI and animal health (Driehuis et al., 2018), thereby exacerbating the challenges facing high-risk cattle. Unfortunately, very little data exist regarding silage vs. hay fed to high-risk cattle. Smerchek et al. (2020) used Charolais × Red Angus weaned steers to examine replacing silage with graded levels of grass hay (0%, 10%, and 20% of the dietary DM) in silage-based receiving diets. DMI increased linearly as the percentage of hay inclusion increased, but hay level did not affect ADG. No morbidity or mortality was noted in the 56-d receiving study. Blom et al. (2020) fed oat hay, dampened oat hay (4 parts oat hay:1 part water), or oat silage across 2 yr to Angus and Angus crossbred steers fed soybean hull-based diets during 42-d receiving periods. No differences among treatments were noted for ADG. In the first year, feeding cattle dampened oat hay or silage increased DMI vs. oat hay alone, but in the second year, cattle fed oat hay and dampened oat hay had greater DMI than cattle fed oat silage. Whether newly received cattle have an aversion to silage remains to be determined but adding dry hays to silage-based diets could provide a means of increasing DMI. Given the availability of silage in the industry and its well-established value in the diets of growing and finishing beef cattle, further work is warranted to examine its use with high-risk newly received cattle.

Despite the generally held belief that roughage is an important dietary ingredient in the diets of newly received cattle, our ability to define the optimal concentration of traditional roughage sources and the value of novel sources is limited. Once newly received cattle begin to eat at normal levels, the effects of dietary roughages can be predicted with reasonable accuracy from their nutrient composition. Thus, future research in this area should focus on how roughage level and source affect the health of newly received cattle, with less emphasis on performance effects.

#### Water

Dehydration is an important stress encountered by newly received cattle (Loerch and Fluharty, 1999), and Preston (2007) concluded that water should be offered immediately to newly received cattle. One method to ensure that cattle receive an adequate amount of water would be to orally drench calves at arrival processing. Tomczak et al. (2019) examined oral drenching of high-risk beef cattle with water in two, 56-d receiving experiments. In one study, water therapy tended to increase DMI, but it also tended to increase mortality (*P* = 0.07). In the second study, ADG and final BW tended to increase with water therapy, but as in the first study, mortality increased (*P* = 0.05) with water therapy. Aspiration of water by the calf during drenching might have resulted in negative effects on mortality. In contrast to these findings, Lopez et al. (2018) drenched newly received cattle with water or a low (200 g/L glycerin) or high (400 g/L glycerin) mixture of glycerin with water and noted no differences in performance during a 42-d receiving period. Moreover, no differences were observed in morbidity or mortality because of the drenching protocols. Carey et al. (2017) reported an increased innate immune response when cattle were supplemented glycerin in their drinking water and challenged with lipopolysaccharide (LPS), and supplementing glycerin in an aqueous drench was hypothesized to be a means of rapidly providing energy to allow the animals to mount a stronger immune response, not necessarily to offset dehydration. Further research is needed to assess various means of hydrating newly received calves and to examine additives that might be delivered via an on-arrival drench to improve performance and health.

#### Minerals

In terms of minerals, Duff and Galyean (2007) focused their review on the effects of Cu, Se, and Zn in receiving diets. Over the past 15 yr since their review, the role of trace minerals in the health and management of newly received cattle has been the most active area of nutritional research with newly received calves. Major areas of effort have revolved around the source of trace minerals and the use of injectable trace minerals in newly received cattle, and most experiments have provided a “package” of several trace minerals rather than focusing on the effects of individual trace minerals. Although packaging of multiple trace minerals together might make sense from an industry perspective, this approach makes it virtually impossible to pinpoint the effects of specific trace minerals.

In a 27-d receiving period, Sharman et al. (2008) compared sources of Zn, Cu, Mn (sulfate vs. amino-acid complexes), and Co (carbonate vs. glucoheptonate) in 216 Angus steers (230 kg) sourced from auction markets. Target mineral intakes were 360, 125, 200, and 12 mg/d for Zn, Cu, Mn, and Co, respectively). Concentrate level of the diet was transitioned from approximately 56% during the first week to approximately 86% by day 15 of the receiving period. Receiving period DMI, ADG, G:F, and serum immunoglobulin concentrations were not affected by mineral source, nor did BRD morbidity differ between treatments, averaging 15.3%. Using a similar treatment structure and target mineral intakes to Sharman et al. (2008), Kegley et al. (2012) obtained 288 bull and steer calves from auction markets and received them into grass paddocks where grain-based supplements were fed as a carrier for the mineral treatments during a 42-d receiving period. Ad libitum access to bermudagrass hay was provided in the paddocks and the grain-based supplement increased from 0.9 to 1.8 kg/d over the first 9 to 14 d. ADG for the 42-d period was increased by feeding organic minerals (0.77 vs. 0.66 kg/d), but BRD morbidity was not affected by treatments. Serum Cu and Zn did not differ between treatments, but titers to infectious bovine rhinotracheitis virus tended to be increased on days 14 and 41 by supplementing inorganic trace minerals. Ryan et al. (2015) also compared sulfate and amino acid-chelated sources of Zn, Cu, and Mn and added a comparison to hydroxy forms of the minerals in 350 calves (240 kg BW). Bermudagrass hay was provided ad libitum and target mineral intakes and grain supplement rates were the same as used by Kegley et al. (2012). Neither performance nor BRD morbidity was affected by the mineral source during the 42- to 45-d receiving periods, and plasma Cu and Zn concentrations measured at the end of the receiving period also did not differ among treatments.

Perhaps a longer term of exposure, or different type of exposure, than can be achieved in a short receiving period is necessary to provide benefit from different sources of trace minerals. Marques et al. (2016) evaluated whether fetal programming via feeding different sources of trace minerals to beef cows during pregnancy would affect the subsequent growth and health of their calves. A total of 84 multiparous Angus × Hereford cows (512 kg BW) were assigned to non-supplemented control vs. inorganic and organic mineral source treatments, which were applied by feeding the cows in drylot during the third trimester of pregnancy. Targeted mineral intakes for supplemented cows were the same as those used by Sharman et al. (2008) and Kegley et al. (2012). Calves were placed in a preconditioning program after weaning for approximately 45 d, followed by a growing and finishing period. Cow performance was not affected by treatment, but liver Zn, Cu, and Co concentrations were greater on day 75 of the treatment period for supplemented vs. control cows. Liver Co was greater on day 75 for cows supplemented with organic mineral, whereas liver Cu was less with the organic vs. the inorganic treatment. Liver concentrations of Cu, Zn, and Co were greater for calves from supplemented cows than from control cows. Weaning weight was increased vs. control by supplementation of organic trace minerals, but it did not differ between organic and inorganic treatments. ADG did not differ among treatments during the growing and finishing periods, but the percentage of calves treated for BRD was less for calves from cows fed the organic minerals vs. calves from cows fed control and inorganic minerals.

In a recent follow-up study, Harvey et al. (2021) fed either inorganic or organic sources of Co, Cu, Mn, and Zn at the same level as used in the Marques et al. (2016) study, but supplementation of cows was started earlier in pregnancy (day 117 vs. the start of the third trimester), but a non-supplemented control was not included in the study. Weaned calves were preconditioned for 45 d, after which heifer calves were managed to assess puberty status, whereas steer calves were backgrounded on pasture for 56 d and then sent to a commercial feedlot for finishing. Even though ADG did not differ between treatments, heifers from cows fed organic minerals reached puberty sooner than heifers from cows fed inorganic minerals. Treatments did not affect feedlot health and performance or carcass characteristics of steer calves.

Organic sources of other trace minerals besides the packaged products that contain Co, Cu, Mn, and Zn have not been extensively studied in the past 15 yr. Sgoifo Rossi et al. (2017) compared sodium selenite and selenium yeast as supplements (0.32 mg/kg of DM) in a 54-d receiving period with 375-kg bullocks imported from France to Italy. ADG, DMI, and feed conversion were improved, and BRD morbidity during the 54-d period was less with selenium yeast vs. sodium selenite.

Based on limited data, Duff and Galyean (2007) did not address the potential role of supplemental Cr in receiving diets. Two studies with newly received calves have been conducted during the past decade involving supplemental chromium propionate. Bernhard et al. (2012) used 180 crossbred beef steers (230 kg) in a 56-d receiving study to evaluate the effects of chromium propionate (0.1, 0.2, and 0.3 mg/kg) vs. a non-supplemented control diet. Daily gain and DMI increased linearly with increasing Cr concentration for the first 28 d of the study, and ADG and G:F increased linearly for the overall 56-d period. The 0.3 mg/kg diet resulted in the lowest BRD morbidity (7.5% vs. 25.9% for the control diet). Smock et al. (2020) compared the effects of a *Bacillus subtilis* PB6 direct-fed microbial and 450 ppb chromium propionate or the combination of the two with a control diet in 384 bull and steer calves (220 kg BW) during a 56-d receiving period. The direct-fed microbial, alone or in combination with Cr, increased DMI for the receiving period compared with other treatments, which was associated with increased ADG. Supplemental Cr did not affect receiving period performance, but both the direct-fed microbial and Cr decreased receiving period BRD morbidity. In a subsequent feedlot finishing period, supplemental Cr decreased ADG and hot carcass weight, but effects of the direct-fed microbial were nonsignificant. These studies show promise for Cr to affect BRD morbidity, but more research is needed.

Given that the nutritional history of newly received cattle is often unknown, approaches that could have a rapid effect on nutritional status for individual calves are of practical interest. Although the magnitude of response could be related to the animal’s trace mineral status, injectable trace minerals can more rapidly increase status than dietary supplementation (Genther and Hansen, 2014). Perhaps, for this reason, the use of injectable trace minerals in newly received calves has received considerable attention over the past decade.

Richeson and Kegley (2011) compared two injectable trace mineral products (1 mL/45.5 kg) with a negative control treatment in a 55-d receiving study. The TM1 product provided Zn, Mn, Cu, and Se at concentrations of 20, 20, 10, and 5 mg/mL, respectively, whereas the TM2 product provided 48, 10, 16, and 5 mg/mL, respectively, of the same four minerals. For the 55-d period, both trace mineral products increased ADG, DMI, and G:F vs. the control, and BRD morbidity was least for the TM1 product vs. control and intermediate for the TM2 product, resulting in the total antibiotic cost per calf being decreased by the two products. Arthington et al. (2014) demonstrated that administering 1 mL of an injectable trace mineral solution (60, 10, 15, and 5 mg/mL of Zn, Mn, Cu, and Se, respectively) to Brangus-crossbred beef calves at 100 and 200 d of age increased liver concentrations of Cu and Se compared with controls that received saline only. In a subset of 24 heifers selected from the original group of calves, heifers that received the injectable trace mineral (5 mL) before weaning and after a 1,600-km transport had greater concentrations of liver Cu, Se, and Zn and acute phase proteins than control heifers but lower ADG. In weaned heifers given 2.5 mL of the injectable trace mineral vs. a saline control on days 0, 51, and 127 of a 177-d development period, humoral immune response to porcine red blood cells was greater following the day 51 treatment in heifers vs. the control.

One key finding with injectable trace minerals is that when used in conjunction with vaccination, they could improve vaccine titer responses. Palomares et al. (2016) reported that administering injectable trace mineral (1 mL/45.5 kg BW; 15, 60, 10, and 5 mg/mL of Cu, Zn, Mn, and Se) to dairy bull calves (3.5 mo of age) enhanced the humoral and cell-mediated responses to a modified live respiratory disease vaccine and increased liver Se, Cu, and Mn concentrations at various sampling times up to 56 d after vaccination. Similarly, Bittar et al. (2020) compared unvaccinated 7-mo-old beef calves with vaccinated calves that received saline or injectable trace mineral (1 mL/45 kg BW; 15, 60, 10, and 5 mg/mL of Cu, Zn, Mn, and Se) at the time of vaccination with a modified-live vaccine for respiratory disease. Five days after vaccination, calves were given an intranasal challenge of bovine viral diarrhea virus type 2. Injectable trace minerals in combination with vaccination increased the humoral immune response to bovine viral diarrhea virus types 1 and 2. Roberts et al. (2016) administered injectable trace minerals (15, 60, 10, and 5 mg/mL of Cu, Zn, Mn, and Se; 2.2 mL/100 kg BW) on arrival vs. a negative control to 275-kg bull and steer calves sourced from an auction market in south Texas. All calves received arrival metaphylaxis with tilmicosin. Treatments did not differ in performance or BRD morbidity during the 42-d receiving period, but bovine viral diarrhea virus-1 antibody titer was greater for calves given injectable trace mineral than for control calves 14 d after arrival vaccination.

Duff and Galyean (2007) concluded that adding trace minerals to diets beyond a level required to compensate for decreased feed intake was difficult to justify. Similarly, these authors concluded that relative to organic vs. inorganic sources of minerals, effects seemed too variable to recommend feeding specific sources. Based on the data available since that time, we would stand by those conclusions in terms of dietary fortification. Although injectable trace minerals could provide a method to address known or unknown trace mineral deficiencies, more research is needed; however, the potential of injectable trace minerals to enhance vaccine titer responses seems strong and deserves further research. The potential role of mineral supplementation of cows to affect the health and performance of their offspring is an area that needs further research. The lack of non-supplemented controls in many studies involving mineral source or route of supplementation, as well as packaging of several minerals into one “treatment,” makes clear-cut interpretation of data in this area challenging.

#### Vitamins

The role of vitamins in the health and performance of newly received calves has not been a major area of work in the past 15 yr. Duff and Galyean (2007) concluded that providing supplemental vitamin E to newly received cattle seemed effective in decreasing BRD morbidity, although effects on performance were less clear, but the industry seems to have adopted higher concentrations of vitamin E in receiving diets (Samuelson et al., 2016). Deters and Hansen (2019a) used 204, single-source Angus-based calves that had been transported 7.5 h to evaluate receiving diets with no supplemental vitamin E, low, medium, and high levels of vitamin E (151, 484, and 995 IU per steer daily). The level of supplemental vitamin E did not affect ADG, DMI, or G:F during the 27-d receiving period, and treatments did not affect the percentage of calves treated for BRD, which averaged 6.8%. By the end of the receiving period, serum and liver concentrations of α-tocopherol increased linearly in response to increasing vitamin E intake, as did antibody titers to bovine viral diarrhea virus type 1; titers to bovine viral diarrhea virus type 2 were not affected by vitamin E.

As with trace minerals, injectable vitamins could offer the potential to quickly alter the status of deficient or marginally deficient cattle. Urban-Chmiel et al. (2011) used 30, 6-wk-old Simmental calves (100 to 120 kg) to compare injections of vitamin E (750 IU) and vitamin C (2.5 g per animal) with controls that received no injections. Leukocytes isolated from the calves were evaluated for sensitivity to *M. haemolytica* leukotoxin. Leukocytes from calves given either vitamin E or C were less sensitive to leukotoxin than controls, with the effect being more consistent with vitamin E. Cusack et al. (2008) compared various combinations and doses of injectable vitamins A, D, and E with an oil-based carrier in 2,465 cattle (340 kg) received at a commercial feedlot. Neither performance nor BRD morbidity was affected by injectable vitamins. In another experiment, 176 cattle were given 5 g of injectable vitamin C at the time of treatment for BRD vs. no vitamin C injection (Cusack et al., 2008). Mortality was less for cattle given vitamin C at the time of BRD treatment (11 vs. 23 deaths per 100 animals) compared with non-injected controls. Deters and Hansen (2020) compared vitamin C injections (5 g per steer) before and after transit for 18 h with a saline control in 72 Angus-based steer calves (356 kg). Injecting vitamin C before transportation increased ADG for the 57-d study, and both pre- and post-transit vitamin C injections resulted in greater DMI vs. control steers for the 57-d period.

Overall, there seems to be little justification for altering the conclusion of Duff and Galyean (2007) with respect to the potential value of vitamin E on BRD morbidity. The role of route of vitamin supplementation (injection vs. feed) deserves further study, as does the potential value of injectable vitamin C for newly received cattle.

#### Probiotics and prebiotics

Yeasts and direct-fed microbials have been used to enhance the performance of beef cattle for several years (McAllister et al., 2011). de Vrese and Schrezenmeir (2008) described prebiotics as selectively fermented ingredients that allow changes in gut microflora (yeasts), and probiotics as viable microorganisms that reach the intestine and have positive health effects (direct-fed microbials). Despite the widespread use of these products, data regarding their use have been variable.

In early weaned calves (not commingled and not high-risk) grazing cool-season annual pastures, Vendramini and Arthington (2007) noted no effects of yeast products on performance, either during grazing or after a 30-d feedlot receiving period. Deters and Hansen (2019b) used newly weaned cattle from a single source to determine whether levels (12 or 18 g per animal daily) or timing (18 g per animal daily fed 19 d after arrival) of a *Saccharomyces cerevisiae* fermentation product affected performance and antioxidant markers. Feeding 12 g/d resulted in greater ADG and G:F from days 0 to 14, but no other differences were found. The 12 g/d dose also resulted in a tendency (*P* = 0.06) for greater concentrations of total, oxidized, and reduced liver glutathione, indicating greater antioxidant capacity. Smith et al. (2020) fed 10 g per animal daily of active yeast (*S. cerevisiae*) to low-risk calves vs. a control with no yeast and noted a greater 47-d ADG and BW with yeast; however, for the remainder of the study, no differences were observed.

The lack of consistent responses to probiotics noted in low-risk cattle might be the result of the cattle not facing challenges that could alter their ruminal environment. These challenges include stress associated with transport and fasting that can cause marked changes to ruminal bacteria and function (Loerch and Fluharty, 1999), resulting in decreased intake. In addition, hypophagia can occur during periods of immune challenge (Brown and Bradford, 2021). Ruminal microflora can affect the host immune system (Zeineldin et al., 2018), and in humans, there is growing evidence that a gut-lung relationship exists, where the gut microbiome may affect lung health (Anand and Mande, 2018). Thus, perhaps because of impaired ruminal function that often occurs in high-risk cattle, probiotics and prebiotics could be beneficial.

Young et al. (2017) fed three yeast cell wall combinations and two concentrations of one strain to beef heifers from two sources. All yeasts were derived from *S. cerevisiae* and were different strains (A, B, or C) or concentrations of one strain (strain A fed daily at 2.5 or 5.0 g per heifer). Although some effects were noted on ADG among the strains, BRD morbidity was not affected by treatment. Finck et al. (2014) used auction barn-sourced cattle to examine the effects of yeast, yeast cell wall, or a combination of both on the health and performance of beef steers. DMI was increased from days 14 to 28 using yeast or cell wall, and overall receiving period DMI was increased using either yeast, cell wall, or the combination compared with a non-supplemented control. No differences were detected among treatments for BRD morbidity. A subset of steers selected from each treatment group was subjected to an LPS challenge, with the result that calves fed yeast or cell wall had lower rectal temperatures and lower serum cortisol after the LPS challenge compared with controls.

Word et al. (2019) fed a combination of *S. cerevisiae* live yeast and yeast cell wall vs. a non-supplemented control to heifers for 31 d before an intratracheal challenge of *M. haemolytica* to measure metabolic and acute-phase responses. Following the challenge, yeast supplementation tended (*P* = 0.06) to decrease nasal lesions and increase water intake, with no other differences observed. Colombo et al. (2021) compared an additive comprised of a yeast-derived probiotic and *B.subtilis* probiotic to inclusion of monensin + tylosin or monensin plus the yeast and *B. subtilis* additive, all of which were fed in a 45-d receiving period. Final pen BW was increased with the addition of the yeast-derived and *B.subtilis* additive and the monensin + yeast-derived and *B. subtilis* additive compared with the monensin + tylosin treatment. In addition, feeding yeast-derived and *B.subtilis* additive resulted in greater DMI for the first 3 wk of the receiving period compared with the other treatments containing monensin. No differences in the percentage of cattle treated for BRD were noted among treatments; nonetheless, the percentage of cattle requiring a second antimicrobial treatment was less for cattle fed yeast-derived and *B.subtilis* additive and the monensin + yeast-derived and *B. subtilis* additive than in the monensin + tylosin treatment.

Rivera et al. (2019) fed a combination of yeast products (*S. cerevisiae* + yeast cell wall) as a supplement to newly received vs. non-supplemented control cattle grazing warm-season perennial pastures and reported a tendency (*P* = 0.08) for greater antimicrobial treatment success for cattle fed yeast products. Moreover, a tendency (*P* < 0.10) was noted for the yeast product to increase BW and ADG over a 56-d period. Because of the low intake at the initiation of the study, the correct dose of yeast products was not achieved until after day 7, which might have delayed the potential positive effects of the yeast treatment. Ponce et al. (2012a) compared a yeast culture and enzymatically hydrolyzed yeast extract product with a carrier-only control in newly received beef heifers. Heifer ADG for the overall 35-d period tended to be greater for cattle fed yeast culture and hydrolyzed yeast, and DMI was increased, with a tendency (*P* = 0.09) for decreased BRD morbidity for cattle fed the yeast product. In single-source, newly weaned cattle, Ogunade et al (2020) fed yeast cell wall products (mannan + glucan) and noted increased DMI and tendencies for increased BW (*P* = 0.07) and ADG (*P* = 0.06) in a 42-d receiving period. Moreover, feeding mannan + glucan increased the expression of five genes related to the immune response to microbial pathogens at day 42.

Keyser et al. (2007) fed yeast (*S. cerevisiae* subspecies *boulardii*) vs. control in a 35-d receiving period to three loads of heifers that were administered florfenicol as a metaphylaxis. The yeast did not affect performance, but it decreased BRD morbidity. In another 35-d experiment (Keyser et al., 2007) in which heifers received the yeast supplement vs. control but were not given metaphylaxis on arrival, treatments did not affect performance or BRD morbidity. Palmer et al. (2019) fed control vs. brewer’s yeast or a yeast culture to newly received beef steers and found no difference in performance or morbidity. A high percentage of their cattle were considered morbid 5 d after arrival, indicating a high degree of stress that might have affected response to the treatments.

Overall, the use of yeasts and other probiotics with higher-risk cattle has resulted in variable responses. As with all other aspects of high-risk cattle studies, results are likely influenced by the degree of morbidity and stress encountered by the animals in each experiment. In a recent meta-analysis, Batista et al. (2022) examined 33 experiments using yeast products fed to beef cattle and found no effect of yeast product on the risk ratio for morbidity or mortality with low heterogeneity. Despite the lack of effect on health measures, Batista et al. (2022) noted that concentrations of acute-phase proteins were greater when yeast products were fed, which is consistent with the results of Kim et al. (2011), who noted that calves fed yeast supplements had greater haptoglobin after vaccination compared with controls. Effects on acute-phase proteins support the hypothesis that if these products are given before immune challenges, better animal responses might be observed. Although challenging to model in a research setting, further work needs to be conducted examining the use of these products before the stresses of weaning, marketing, and receiving occur. Moreover, research on the use of oral pastes or drenches could be a promising way to deliver these products to the animal when low feed intake is a challenge.

### Other Additives and Approaches

As noted previously, there is increasing scrutiny involving the use of antimicrobial pharmaceuticals in livestock production (FDA, 2018). As a result, various compounds and additives have been evaluated regarding their effects on animal health and performance, with the goal of decreased antibiotic use. Certain essential oils seem to have the potential to modify ruminal fermentation (Khiaosa-ard and Zebeli, 2013), which might in turn improve overall animal performance and health. In lower risk Charolais cattle, feeding the essential oils cinnamaldehyde, eugenol, and capsicum resulted in greater antibody production (*P* < 0.05) and serum bactericidal activity (Compiani et al., 2013). Little work with essential oils, however, has been done with high-risk cattle. de Souza et al. (2018) fed plant extracts in combination with sodium saccharin to beef calves (197 kg) and noted no effects on performance or health. de Sousa et al. (2019) fed saponins at the rate of 1 or 2 g daily in a 59-d receiving period using 220-kg beef calves. Saponin fed at the rate of 2 g per animal daily significantly increased ADG and improved gain efficiency. Moreover, feeding either 1 or 2 g per animal daily resulted in a greater success rate for the initial BRD therapy and fewer antibiotic treatments compared with a control group receiving no saponins. Using compounds that replicate the bovine appeasing pheromone to potentially improve health and performance was examined in a 45-d receiving period (Columbo et al., 2020). The compound resulted in greater ADG and gain efficiency and fewer antibiotic treatments compared with cattle not given the compound. Although limited published work has been done with these various additives, available data suggest that they deserve further investigation.

Daigle et al. (2018) evaluated the effects of exercise on the performance, behavior, and health of beef cattle. Exercise decreased gains during the receiving period without improving animal health or altering animal behavior. Likewise, Woolsoncroft et al. (2018) reported that exercise had minimal effects on calf health but suggested that exercise could potentially improve feed efficiency in newly received beef calves.

## Summary and Research Needs

**Figure 1.**
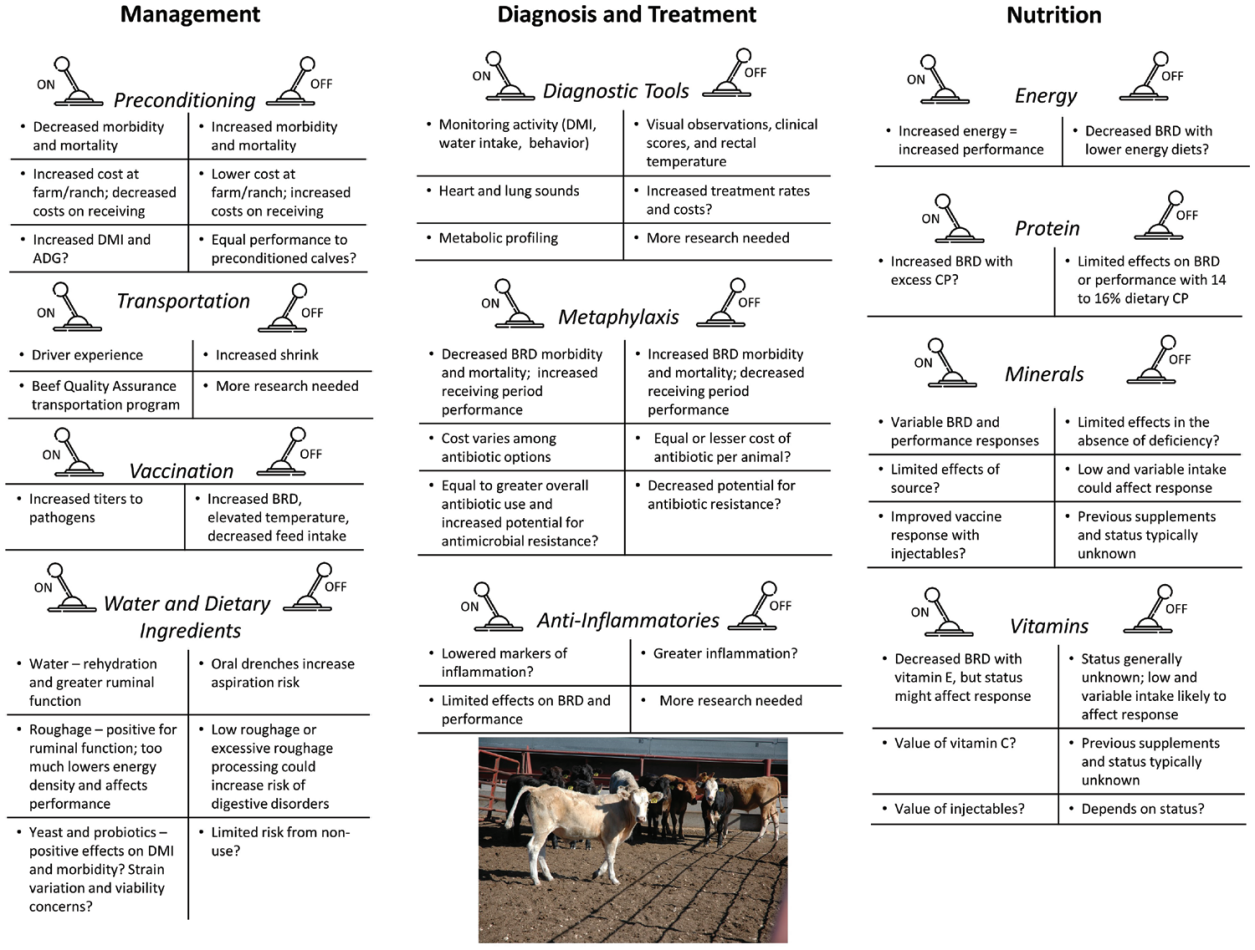
Control levers available to beef producers to manage the health and performance of newly received beef calves.

The various areas of research addressed in this review are summarized in Figure 1 in the context of control levers: “on/off” options for implementation by producers. Many of the items listed under these on/off switches are followed by a statement that more research is needed or by a question mark, suggesting that these are areas that need further study.

The value of practices like preconditioning, vaccination, and metaphylaxis is well-established, but even within these areas, important research remains to be conducted (e.g., effects of metaphylaxis on AMR). Indeed, with concerns about AMR, non-antimicrobial approaches to prevent or decrease the incidence of BRD will continue to be an area in which we need to invest research resources. Accurate diagnosis of BRD is a significant practical challenge. Continued work on the development of real-time diagnostic tools that can be used to identify and treat cattle with BRD will be an important area of research. In addition, developing tools for early identification of BRD (remote monitoring related to behavior or physiological measurements, etc.) deserves significant attention. Although our knowledge of the nutritional requirements of newly received cattle has advanced significantly, there are still many areas where a greater understanding is needed. Efforts to rapidly assess the nutritional status of newly received calves for protein, vitamins, and minerals and to apply targeted supplementation programs could prove valuable, and the effects of cow nutrition on calf health is an area of research that merits emphasis. With a focus on these and other areas of research noted throughout the text of this review, we are confident that the next 15 yr will yield significant advances in our knowledge of the nutrition and health of newly received cattle and provide important practical management tools for the beef cattle industry.

## Abbreviations

ADG: average daily gain
AMR: antimicrobial resistance
BRD: bovine respiratory disease
BW: body weight
DMI: dry matter intake
G:F: gain-to-feed ratio
NE: net energy

## References

[CIT0001] Abell, K. M., M. E. Theurer, R. L. Larson, B. J. White, and M. Apley. 2017. A mixed treatment comparison meta-analysis of metaphylaxis treatments for bovine respiratory disease in beef cattle. J. Anim. Sci. 95:626–635. doi:10.2527/jas.2016.1062 28380607

[CIT0002] Anand, S., and S. S. Mande. 2018. Diet, microbiota and gut-lung connection. Front. Microbiol. 9:2147. doi:10.3389/fmicb.2018.02147 30283410 PMC6156521

[CIT0003] Anderson, J. E., R. S. Walker, K. Harborth, M. Garcia, and C. C. Williams. 2016. Effects of weaning method and preconditioning period on calf performance, haptoglobin concentrations, feedlot health and performance, and carcass traits. Prof. Anim. Sci. 32:279–286. doi:10.15232/pas.2015-01442

[CIT0004] Arthington, J. D., P. Moriel, P. G. Martins, G. C. Lamb, and L. J. Havenga. 2014. Effects of trace mineral injections on measures of performance and trace mineral status of pre- and postweaned beef calves. J. Anim. Sci. 92:2630–2640. doi:10.2527/jas.2013-7164 24867937

[CIT0005] Bailey, E. A., J. R. Jaeger, J. W. Waggoner, G. W. Preedy, L. A. Pacheco, and K. C. Olson. 2016. Effect of fence-line or drylot weaning on the health and performance of beef calves during weaning, receiving, and finishing. Prof. Anim. Sci. 32:220–228. doi:10.15232/pas.2015-01456

[CIT0006] Ball, J. J., E. B. Kegley, J. Sarchet, and J. G. Powell. 2019. Comparison of treatment protocols for bovine respiratory disease in high-risk, newly received beef calves. Appl Anim. Sci. 35:278–283. doi:10.15232/aas.2018-01836

[CIT0007] Batista, L. H. C., I. A., Cidrini, L. F. Prados, A. A. C. Cruz, J. A. Torrecilhas, G. R. Siquieira, and F. D. Resende. 2022. A meta-analysis of yeast products for beef cattle under stress conditions: performance, health, and physiological parameters. Anim. Sci and Feed Tech. 283:115182. doi:10.1016/j.anifeedsci.2021.115182

[CIT0008] Bernhard, B. C., N. C. Burdick, W. Rounds, R. J. Rathmann, J. A. Carroll, D. N. Finck, M. A. Jennings, T. R. Young, and B. J. Johnson. 2012. Chromium supplementation alters the performance and health of feedlot cattle during the receiving period and enhances their metabolic response to a lipopolysaccharide challenge. J. Anim. Sci. 90:3879–3888. doi:10.2527/jas.2011-4981 22665638

[CIT0009] Berry, B. A., C. R. Krehbiel, A. W. Confer, D. R. Gill, R. A. Smith, and M. Montelongo. 2004. Effects of dietary energy and starch concentrations for newly received feedlot calves: I. Growth performance and health. J. Anim. Sci. 82:837–844. doi:10.2527/2004.823837x 15032441

[CIT0010] Bewley, J. M., M. E. Einstein, M. W. Grott, and M. M. Schutz. 2008. Comparison of reticular and rectal core body temperatures in lactating dairy cows. J. Dairy Sci. 91:4661–4672. doi:10.3168/jds.2007-0835 19038942

[CIT0011] Bittar, J. H. J., R. A. Palomares, D. J. Hurley, A. Hoyos-Jaramillo, A. Rodriguez, A. Stoskute, B. Hamrick, N. Norton, M. Adkins, J. T. Saliki, et al. 2020. Immune response and onset of protection from bovine viral diarrhea virus 2 infection induced by modified-live virus vaccination concurrent with injectable trace minerals administration in newly received beef calves. Vet. Immunol. Immunopathol. 225:110055. doi:10.1016/j.vetimm.2020.110055 32438245 PMC7184996

[CIT0012] Blakebrough-Hall, C., A. Dona, M. J. D’occhio, J. McMeniman, and L. A. González. 2020a. Diagnosis of bovine respiratory disease in feedlot cattle using blood ^1^H NMR metabolomics. Sci. Rep. 10:115. doi:10.1038/s41598-019-56809-w 31924818 PMC6954258

[CIT0013] Blakebrough-Hall, C., J. P. McMeniman, and L. A. Gonzalez. 2020b. An evaluation of the economic effects of bovine respiratory disease on animal performance, carcass traits, and economic outcomes in feedlot cattle defined using four BRD diagnosis methods. J. Anim. Sci. 98(4):1–11. doi:10.1093/jas/skaa005 PMC6996507 31930299

[CIT0014] Blom, E. J., W. W. Gentry, R. H. Pritchard, and K. E. Hales. 2020. Evaluation of inclusion of hay, dampened hay, and silage in receiving diets of newly weaned beef calves. Appl. Anim. Sci. 36:367–376. doi:10.15232/aas.2019-01922

[CIT0015] Boyles, S. L., S. C. Loerch, and G. D. Lowe. 2007. Effects of weaning management strategies on performance and health of calves during feedlot receiving. Prof. Anim. Sci. 23:637–641. doi:10.15232/S1080-7446(15)31034-2

[CIT0016] Brooks, J. M., J. J. Randall, and G. C. Duff. 2021. Effects of preconditioning on the nasopharyngeal microbiota of beef calves grazing winter wheat. Transl. Anim. Sci. 5:S11–S15. doi:10.1093/tas/txab192

[CIT0017] Brooks, K. R., K. C. Raper, C. E. Ward, B. P. Holland, C. R. Krehbiel, and D. L. Step. 2011. Economic effects of bovine respiratory disease on feedlot cattle during backgrounding and finishing phases. Prof. Anim. Sci. 27:195–203. doi:10.15232/S1080-7446(15)30474-5

[CIT0018] Brown, W. E., and B. J. Bradford. 2021. Invited review: mechanisms of hypophagia during disease. J. Dairy Sci. 104:9418–9436. doi:10.3168/jds.2021-20217 34099296

[CIT0019] Cameron, A., and T. A. McAllister. 2016. Antimicrobial usage and resistance in beef production. J. Anim. Sci. Biotechnol. 7:68. doi:10.1186/s40104-016-0127-3 27999667 PMC5154118

[CIT0020] Carey, R. E., K. L. Samuelson, E. R. Oosthuysen, F. A. Lopez, S. L. Pillmore, L. T. Klump, N. C. Burdick-Sanchez, J. A. Carroll, J. A. Hernandez-Gifford, and C. A. Loest. 2017. Glycerin supplementation via drinking water alters nitrogen balance and immune response of beef steers during and endotoxin challenge. Proc. West Sect. Amer. Soc. Anim. Sci. 68:70–75. doi:10.2527/asasws.2017.0024

[CIT0021] Cernicchiaro, N., B. J. White, D. G. Renter, A. H. Babcock, L. Kelly, and R. Slattery. 2012. Effects of body weight loss during transit form sale barns to commercial feedlots on health and performance in feeder cattle cohorts arriving to feedlots from 2000 to 2008. J. Anim. Sci. 90:1940–1947. doi:10.2527/jas2011-4600 22247120

[CIT0022] Checkley, S. L., J. R. Campbell, M. Chirino-Trejo, E. D. Janzen, and C. L. Waldner. 2010. Associations between antimicrobial use and the prevalence of antimicrobial resistance in fecal *Escherichia coli* from feedlot cattle in western Canada. Can. Vet J. 51:853–861. PMID: 21037885. 21037885 PMC2905004

[CIT0023] Coetzee, J. F., D. R. Magstadt, P. K. Sidhu, L. Follett, A. M. Schuler, A. C. Krull, V. L. Cooper, T. J. Engelken, M. D. Kleinhenz, and A. M. O’Connor. 2019. Association between antimicrobial drug class for treatment and retreatment of bovine respiratory disease (BRD) and frequency of resistant BRD pathogen isolation from veterinary diagnostic laboratory samples. PLoS One 14:e0219104. doi:10.1371/journal.pone.0219104 31835273 PMC6910856

[CIT0024] Colombo, E. A., R. F. Cooke, A. P. Brandão, J. B. Wiegand, K. M. Schubach, C. A. Sowers, G. C. Duff, E. Block, and V. N. Gouvêa. 2021. Performance, health, and physiological responses of newly received feedlot cattle supplemented with pre- and probiotic ingredients. Animal 15:100214. doi:10.1016/j.animal.2021.100214 34029789

[CIT0025] Colombo, E. A., R. F. Cooke, A. P. Brandão, J. B. Wiegand, K. M. Schubach, C. Sowers, G. C. Duff, V. N. Gouvêa, and B. I. Cappellozza. 2020. Administering an appeasing substance to optimize welfare and performance of receiving cattle. Transl. Anim. Sci. 4(Suppl 1):S1–S5. doi:10.1093/tas/txaa086 33381712 PMC7754225

[CIT0026] Compiani, R., C. A. Sgoifo-Rossi, A. Pizzi, and V. Dell’Orto. 2013. Administration of essential oils cinnamaldehyde, eugenol, and capsicum to beef cattle: effects on health status and growth performance. In: Boiti, C., A. Ferlazzo, A. Gaiti, and A. Pugliese, editors. Trends in veterinary sciences, Chapter 32. Berlin (Heidelberg): Springer. doi:10.1007/978-3-642-36488-4_32

[CIT0027] Cooke, R. F. 2017. Invited paper: nutritional and management considerations for beef cattle experiencing stress-induced inflammation. Prof. Anim. Sci. 33:1–11. doi:10.15232/pas.2016-01573

[CIT0028] Cooke, R. F., B. I. Cappellozza, T. A. Guarnieri Filho, and D. W. Bohnert. 2013a. Effects of flunixin meglumine administration on physiological and performance responses of transported feeder cattle. J. Anim. Sci. 91:5500–5506. doi:10.2527/jas.2013-6336 24045487

[CIT0029] Cooke, R. R., T. A. Guarnieri Filho, B. I. Cappellozza, and D. W. Bohnert. 2013b. Rest stops during road transport: impacts on performance and acute-phase protein responses of feeder cattle. J. Anim. Sci. 91:5448–5454. doi:10.2527/jas2013-6357 23989870

[CIT0030] Cusack, P. M., N. P. McMeniman, and I. J. Lean. 2008. Effects of injectable vitamins A, D, E and C on the health and growth rate of feedlot cattle destined for the Australian domestic market. Aust. Vet. J. 86:81–87. doi:10.1111/j.1751-0813.2008.00255.x 18304043

[CIT0031] Daigle, C. L., A. J. Mathias, E. E. Ridge, R. Gill, T. A. Wickersham, and J. W. Sawyer. 2018. Case study: effect of exercise programs during receiving in a commercial feedlot on behavior and productivity of Brahman crossbred calves: results from a commercial environment and a comparison to the research environment. Prof. Anim. Sci. 34:653–663. doi:10.15232/pas.2018-01744

[CIT0032] Dennis, E. J., T. C. Schroeder, D. G. Renter, and D. L. Pendell. 2018. Value of arrival metaphylaxis in U.S. cattle industry. J. Agric. Resour. Econ. 43:233–250. doi:10.22004/ag.econ.273448

[CIT0033] Deters, E. L., and S. L. Hansen. 2019a. Vitamin E supplementation strategies during feedlot receiving: effects on beef steer performance, antibody response to vaccination, and antioxidant defense. J. Anim. Sci. 97:4362–4369. doi:10.1093/jas/skz289 31504608 PMC6776265

[CIT0034] Deters, E. L., and S. L. Hansen. 2019b. Effect of supplementing a *Saccharomyces cerevisiae* fermentation product during a preconditioning period prior to transit on receiving period performance, nutrient digestibility, and antioxidant defense by beef steers. Transl. Anim. Sci. 3:1227–1237. doi:10.1093/tas/txz140 32704886 PMC7200456

[CIT0035] Deters, E. L., and S. L. Hansen. 2020. Pre-transit vitamin C injection improves post-transit performance of beef steers. Animal 14:2083–2090. doi:10.1017/S1751731120000968 32381151 PMC7503131

[CIT0036] de Sousa, O. A., R. F. Cooke, A. P. Brandão, K. M. Schubach, T. F. Schumaher, D. W. Bohnert, and R. S. Marques. 2019. Productive and physiological responses of feeder cattle supplemented with *Yucca schidigera* extract during feedlot receiving. J. Anim. Sci. 97:208–219. doi:10.1093/jas/sky412 30351413 PMC6313122

[CIT0037] de Souza, K. A., R. F. Cooke, K. M. Schubach, A. P. Brandão, T. F. Schumaher, I. N. Prado, R. S. Marques, and D. W. Bohnert. 2018. Performance, health and physiological responses of newly weaned feedlot cattle supplemented with feed-grade antibiotics or alternative feed ingredients. Animal 12:2521–2528. doi:10.1017/S1751731118000551 29576039

[CIT0038] de Vrese M., and J. Schrezenmeir. 2008. Probiotics, prebiotics, and symbiotics. In: Stahl, U., U. E. Donalies, and E. Nevoigt, editors. Food biotechnology. Advances in biochemical engineering/biotechnology. Vol 111. Berlin (Heidelberg): Springer. doi:10.1007/10_2008_097 18461293

[CIT0039] Doster, E., P. Rovira, N. R. Noyes, B. A. Burgess, X. Yang, M. D. Weinroth, S. M. Lakin, C. J. Dean, L. Linke, R. Magnuson, et al. 2018. Investigating effects of tulathromycin metaphylaxis on the fecal resistome and microbiome of commercial feedlot cattle early in the feeding period. Front. Microbiol. 9:1715. doi:10.3389/fmicb.2018.01715 30105011 PMC6077226

[CIT0040] Driehuis, F., J. M. Wilkinson, Y. Jiang, I. Ogunade, and A. T. Adesogan. 2018. Silage review: animal and human health risks from silage. J. Dairy Sci. 101:4093–4110. doi:10.3168/jds.2017-13836 29685279

[CIT0041] Duff, G. C., and M. L. Galyean. 2007. Board-invited review: recent advances in management of highly stressed, newly received feedlot cattle. J. Anim. Sci. 85:823–840. doi:10.2527/jas.2006-501 17085724 PMC7109667

[CIT0042] Farran, T. B., R. D. Hunter, S. P. Montgomery, J. J. Sindt, D. A. Blasi, and J. S. Drouillard. 2001. Using a mixture of cottonseed hulls and cottonseed meal to replace alfalfa hay in diets for stressed feeder calves. Kansas Agric. Exp. Sta., Cattlemen's Day, pp. 95–97.

[CIT0043] FDA. 2018. Supporting antimicrobial stewardship in veterinary settings: goals for fiscal years 2019–2023. FDA Center for Veterinary Medicine. https://www.fda.gov/files/animal%20&%20veterinary/published/Supporting-Antimicrobial-Stewardship-in-Veterinary-Settings--Goals-for-Fiscal-Years-2019-2023.pdf

[CIT0044] Finck, D. N., R. R. B. Ribeiro, N. C. Burdick, S. L. Parr, J. A. Carroll, T. R. Young, B. C. Bernhard, J. R. Corley, A. G. Estefan, R. J. Rathmann, et al. 2014. Yeast supplementation alters the performance and health status of receiving cattle. Prof. Anim. Sci. 30:333–341. doi:10.15232/S1080-7446(15)30125-X

[CIT0045] Galyean, M. L., and M. E. Hubbert. 2014. Review: traditional and alternative sources of fiber-roughage values, effectiveness, and levels in starting and finishing diets. Prof. Anim. Sci. 30:571–584. doi:10.15232/pas.2014-01329

[CIT0046] Genther, O. N., and S. L. Hansen. 2014. A multielement trace mineral injection improves liver copper and selenium concentrations and manganese superoxide dismutase activity in beef steers. J. Anim. Sci. 92:695–704. doi:10.2527/jas.2013-7066 24398829

[CIT0047] González, L. A., K. S. Schwartzkopf-Genswein, M. Bryan, R. Silasi, and F. Brown. 2012. Relationships between transport conditions and welfare outcomes during commercial long haul transport of cattle in North America. J. Anim. Sci. 90:3640–3651. doi:10.2527/jas.2011-4796 22665659

[CIT0048] Gozho, G. N., J. C. Plaizier, D. O. Krause, A. D. Kennedy, and K. M. Wittenberg. 2005. Subacute ruminal acidosis induces ruminal lipopolysaccharide endotoxin release and triggers an inflammatory response. J. Dairy Sci. 88:1399–1403. doi:10.3168/jds.S0022-0302(05)72807-1 15778308

[CIT0049] Guarnieri Filho, T. A., R. F. Cooke, B. I. Cappellozza, M. M. Reis, R. S. Marques, and D. W. Bohnert. 2014. Effects of meloxicam administration on physiological and performance responses of transported feeder cattle. J. Anim. Sci. 92:4137–4144. doi:10.2527/jas.2014-7783 25023798

[CIT0050] Harvey, K. M., R. F. Cooke, E. A. Colombo, B. Rett, O. A. de Sousa, L. M. Harvey, J. R. Russel, K. G. Pohler, and A. P. Brandão. 2021. Supplementing organic-complexed or inorganic Co, Cu, Mn, and Zn to beef cows during gestation: postweaning responses of offspring reared as replacement heifers or feeder cattle. J. Anim. Sci. 99(6):1–11. doi:10.1093/jas/skab082 PMC8186539 33715010

[CIT0051] Holland, B. P., L. O. Burciaga-Robles, D. L. VanOverbeke, J. N. Shook, D. L. Step, C. J. Richards, and C. R. Krehbiel. 2010. Effect of bovine respiratory disease during preconditioning on subsequent feedlot performance, carcass characteristics, and beef attributes. J. Anim. Sci. 88:2486–2499. doi:10.2527/jas.2009-2428 20190167

[CIT0052] Holman, D. B., W. Yang, and T. W. Alexander. 2019. Antibiotic treatment in feedlot cattle: a longitudinal study of the effect of oxytetracycline and tulathromycin on the fecal and nasopharyngeal microbiota. Microbiome 7:86. doi:10.1186/s40168-019-0696-4 31167657 PMC6549328

[CIT0053] Kegley, E. B., M. R. Pass, J. C. Moore, and C. K. Larson. 2012. Supplemental trace minerals (zinc, copper, manganese, and cobalt) as Availa-4 or inorganic sources for shipping-stressed beef cattle. Prof. Anim. Sci. 28:313–318. doi:10.15232/S1080-7446(15)30361-2

[CIT0054] Keyser, S. A., J. P. McMeniman, D. R. Smith, J. C. McDonald, and M. L. Galyean. 2007. Effects of *Saccharomyces cerevisiae* subspecies *boulardii* CNCM I-1079 on feed intake by healthy beef cattle, treated with florfenicol and on health and performance of newly received beef heifers. J. Anim. Sci. 85:1264–1273. doi:10.2527/jas.2006-751 17264239

[CIT0055] Khiaosa-ard, R., and Q. Zebeli. 2013. Meta-analysis of the effects of essential oils and their bioactive compounds on rumen fermentation characteristics and feed efficiency in ruminants. J. Anim. Sci. 91:1819–1830. doi:10.2527/jas.2012-5691 23345564

[CIT0056] Kim, M., J. Seo, C. Yun, S. Kang, J. Ko, and J. Ha. 2011. Effects of hydrolyzed yeast supplementation in calf starter on immune response to vaccine challenge in neonatal calves. Anim. 6:953–960. doi:10.1017/S175731110002673 22440035

[CIT0057] Lehmkuhler, J. W., and M. S. Kerley. 2007. Blood meal and fish meal as supplements to increase the amino acid to energy ratio in steer receiving diets. Prof. Anim. Sci. 23:253–259. doi:10.15232/S1080-7446(15)30970-0

[CIT0058] Li, P., Y. L. Yin, D. Li, S. W. Kim, and G. Wu. 2007. Amino acids and immune function. Br. J. Nutr. 98:237–252. doi:10.1017/S000711450769936X 17403271

[CIT0059] Lippolis, K. D., R. F. Cooke, K. M. Schubach, A. P. Brandão, L. G. da Silva, R. S. Marques, and D. W. Bohnert. 2016. Altering the time of vaccination against respiratory pathogens to enhance antibody response and performance of feeder cattle. J. Anim. Sci. 94:3987–3995. doi:10.2527/jas.2016-0673 27898919

[CIT0060] Loerch, S. C., and F. L. Fluharty. 1999. Physiological changes and digestive capabilities of newly received feedlot cattle. J. Anim. Sci. 77:1113–1119. doi:10.2527/1999.7751113x 10340577

[CIT0061] Lofgreen, G. P., A. E. El Tayeb, and H. E. Kiesling. 1981. Millet and alfalfa hays alone and in combination with high-energy diets for receiving stressed calves. J. Anim. Sci. 52:959–968. doi:10.2527/jas1981.525959x 7240056

[CIT0062] Lopez, F. A., E. R. Oosthuysen, G. C. Duff, J. T. Richeson, K. L. Samuelson, M. E. Hubbert, and C. A. Löest. 2018. Health, performance, and complete blood counts of newly received feedlot heifers in response to an oral drench of water and crude glycerin. Transl. Anim. Sci. 2(Suppl 1):S74–S78. doi:10.1093/tas/txy028 32704740 PMC7200899

[CIT0063] Loya-Olguin, F., L. Avendaño-Reyes, A. M. Encinias, D. A. Walker, N. A. Elam, and S. A. Soto-Navarro. 2008. Influence of slice baling on feeding value of alfalfa hay in receiving and finishing diets for feedlot cattle. J. Anim. Sci. 86:2749–2755. doi:10.2527/jas.2007-0637 18539841

[CIT0064] Marques, R. S., R. F. Cooke, C. L. Francisco, and D. W. Bohnert. 2012. Effects of twenty-four hour transport or twenty-four hour feed and water deprivation on physiologic and performance responses of feeder cattle. J. Anim. Sci. 90:5040–5046. doi:10.2527/jas.2012-5425 22851237

[CIT0065] Marques, R. S., R. F. Cooke, M. C. Rodrigues, B. I. Cappellozza, R. R. Mills, C. K. Larson, P. Moriel, and D. W. Bohnert. 2016. Effects of organic or inorganic cobalt, copper, manganese, and zinc supplementation to late-gestating beef cows on productive and physiological responses of the offspring. J. Anim. Sci. 94:1215–1226. doi:10.2527/jas.2015-0036 27065282

[CIT0066] Massey, C., K. C. Dhuyvetter, R. V. Llewelyn, and D. A. Blasi. 2011. Castration and morbidity and their effects on performance, carcass quality, and price differentials for bulls and steers. Prof. Anim. Sci. 27:19–28. doi:10.15232/S1080-7446(15)30440-X

[CIT0067] McAllister, T. A., K. A. Beauchemin, A. Y. Alazzeh, J. Baah, R. M. Teather, and K. Stanford. 2011. Review: the use of direct fed microbials to mitigate pathogens and enhance production in cattle. Can. J. Anim. Sci. 91:193–211. doi:10.4141/cjas10047

[CIT0068] Montgomery, S. P., J. J. Sindt, M. A. Greenquist, W. F. Miller, J. N. Pike, E. R. Loe, M. J. Sulpizio, and J. S. Drouillard. 2009. Plasma metabolites of receiving heifers and the relationship between apparent bovine respiratory disease, body weight gain, and carcass characteristics. J. Anim. Sci. 87:328–333. doi:10.2527/jas.2008-0969 18820162

[CIT0069] Munoz, V. I., K. L. Samuelson, D. J. Tomczak, H. A. Seiver, T. M. Smock, and J. T. Richeson. 2020. Comparative efficacy of metaphylaxis with tulathromycin and pentavalent modified-live virus vaccination in high-risk, newly received feedlot cattle. Appl. Anim. Sci. 36:799–807. doi:10.15232/aas.2020-02054

[CIT0070] NAHMS. 2013. National animal health monitoring system. Feedlot 2011. Part IV: health and health management on U.S. feedlots with a capacity of 1000 or more head. Fort Collins (CO): USDA-APHIS-VS-CEAH-NAHMS. Available from https://www.aphis.usda.gov/animal_health/nahms/feedlot/downloads/feedlot2011/Feed11_dr_PartIV_1.pdf

[CIT0071] NASEM. 2016. Nutrient requirements of beef cattle. 8th ed. Washington (DC): National Academy Press. doi:10.17226/19014

[CIT0072] Nautrup, B. P., I. Van Vlaenderen, S. M. Gasper, and R. E. Holland. 2013. Estimating the comparative clinical and economic consequences of tulathromycin for treatment of present or anticipated outbreaks of bovine respiratory disease in feedlot cattle in the United States. J. Anim. Sci. 91:5868–5877. doi:10.2527/jas.2013-6709 24126273

[CIT0073] Nichols, B. 2014. Scoring helps assess bovine respiratory disease. Ardmore (OK): Ag News and Views, Noble Research Institute. Available from https://www.noble.org/globalassets/images/news/ag-news-and-views/2014/12/pdf/brd-scoring.pdf

[CIT0074] Nickell, J., L. Bryant, K. F. Lechtenberg, and C. Cull. 2020. Evaluation of antimicrobial or non-antimicrobial treatments in commercial feedlot cattle with mild bovine respiratory disease based on a refined case-definition. Front. Vet. Sci. 7:571697. doi:10.3389/fvets.2020.571697 33134355 PMC7575691

[CIT0075] Nickell, J. S., J. P. Hutcheson, D. G. Renter, and D. A. Amrine. 2021. Comparison of a traditional bovine respiratory disease control regimen with a targeted program based upon individualized risk predictions generated by the Whisper On Arrival technology. Transl. Anim. Sci. 5:txab081. doi:10.1093/tas/txab081 34222823 PMC8246073

[CIT0076] O’Connor, A. M., D. Hu, S. C. Totton, N. Scott, C. B. Winder, B. Wang, C. Wang, J. Glanville, H. Wood, B. White, et al. 2019. A systematic review and network meta-analysis of injectable antibiotic options for the control of bovine respiratory disease in the first 45 days post arrival at the feedlot. Anim. Health Res. Rev. 20:163–181. doi:10.1017/S1466252320000031 32081117

[CIT0077] Ogunade, I. M., G. Taiwo, M. Estrada-Reyes, J. Yun, A. A. Pech-Cervantes, and S. O. Peters. 2020. Effects of a blend of mannan and glucan on growth, performance, apparent nutrient digestibility, energy status, and whole blood immune gene expression of beef steers during a 42-d receiving period. Transl. Anim. Sci. 5:1–13. doi:10.1093/tas/txaa226 PMC7846145 33542996

[CIT0078] Oosthuysen, R. R., M. E. Hubbert, K. L. Samuelson, E. J. Scholljegerdes, G. C. Duff, and C. A. Löest. 2016. Health evaluation of immune-stimulated and hay-supplemented feedlot receiving calves as assessed by blood gas analysis. Proc West. Sec. Am. Soc. Anim. Sci. 67:83–85.

[CIT0079] Palmer, E. A., E. B. Kegley, J. J. Ball, P. A. Beck, J. A. Hornsby, J. L. Reynolds, B. P. Shoulders, A. R. Boyer, and J. G. Powell. 2019. Influence of commercial yeast products in diets of beef cattle new to the feedlot environment. Appl. Anim. Sci. 35:491–497. doi:10.15232/aas.2019-01847

[CIT0080] Palomares, R. A., D. J. Hurley, J. H. Bittar, J. T. Saliki, A. R. Woolums, F. Moliere, L. J. Havenga, N. A. Norton, S. J. Clifton, A. B. Sigmund, et al. 2016. Effects of injectable trace minerals on humoral and cell-mediated immune responses to bovine viral diarrhea virus, bovine herpes virus 1 and bovine respiratory syncytial virus following administration of a modified-live virus vaccine in dairy calves. Vet. Immunol. Immunopathol. 178:88–98. doi:10.1016/j.vetimm.2016.07.003 27496747

[CIT0081] Peterson, S. J., B. L. Nuttleman, D. B. Burken, M. K. Luebbe, G. E. Erickson, and J. C. MacDonald. 2015. Use of a pelleted corn-residue complete feed in calf receiving diets. Prof. Anim. Sci. 31:201–206. doi:10.15232/pas.2014-01363

[CIT0082] Poe, K. D., P. A. Beck, J. T. Richeson, M. S. Gadberry, E. B. Kegley, T. W. Hess, and D. S. Hubbell. 2013. Effects of respiratory vaccination timing and growth-promoting implant and health, performance, and immunity of high-risk, newly received stocker cattle. Prof. Anim. Sci. 29:413–419. doi:10.15232/S1080-7446(15)30254-0

[CIT0083] Ponce, C. H., J. S. Schutz, C. C. Elrod, U. Y. Anele, and M. L. Galyean. 2012a. Effects of dietary supplementation of a yeast product on performance and morbidity of newly received beef heifers. Prof. Anim. Sci. 28:618–622. doi:10.15232/S1080-7446(15)30419-8

[CIT0084] Ponce, C. H., D. R. Smith, J. S. Schultz, and M. L. Galyean. 2012b. Effects of receiving diets based on wet corn gluten feed on performance and morbidity of newly received beef heifers and in vitro fermentation. Prof. Anim. Sci. 28:213–220. doi:10.15232/S1080-7446(15)30342-9

[CIT0085] Preston, R. L. 2007. Receiving cattle nutrition. Vet. Clin. North Am. Food Anim. Pract. 23:193–205, v–vi. doi:10.1016/j.cvfa.2007.04.001 17606146

[CIT0086] Ratcliff, M. D., E. B. Kegley, J. G. Powell, J. Hawley, K. S. Lusby, M. P. Rowe, S. A. Gunter, L. B. Daniels, and D. S. Hubbell III. 2014. Assessment of the effect of castration upon arrival on long-term growth performance of stocker cattle. Prof. Anim. Sci. 30:466–475. doi:10.15232/pas.2012-01177

[CIT0087] Reinhardt, C. D., M. L. Hands, T. T. Marston, J. W. Waggoner, and L. R. Corah. 2012. Relationships between feedlot health, average daily gain, and carcass traits of Angus steers. Prof. Anim. Sci. 28:11–19. doi:10.15232/S1080-7446(15)30311-9

[CIT0088] Reuter, R. R., J. A. Carroll, J. W. Dailey, B. J. Cook, and M. L. Galyean. 2008. Effects of dietary energy source and level and injection of tilmicosin phosphate on immune function in lipopolysaccharide-challenged beef steers. J. Anim. Sci. 86:1963–1976. doi:10.2527/jas.2007-0838 18407986

[CIT0089] Richeson, J. T. 2020. Behavior assessment and applications for BRD diagnosis: beef. Anim. Health Res. Rev. 21:192–195. doi:10.1017/S1466252320000249 33682665

[CIT0090] Richeson, J. T., P. A. Beck, M. S. Gadberry, S. A. Gunter, T. W. Hess, D. S. Hubbell III, and C. Jones. 2008. Effects of on-arrival versus delayed modified live virus vaccination on health, performance, and serum infectious bovine rhinotracheitis titers of newly received beef calves. J. Anim. Sci. 86:999–1005. doi:10.2527/jas.2007-0593 18192559

[CIT0091] Richeson, J. T., and E. B. Kegley. 2011. Effect of supplemental trace minerals from injection on health and performance of highly stressed, newly received beef heifers. Prof. Anim. Sci. 27:461–466. doi:10.15232/S1080-7446(15)30519-2

[CIT0092] Richeson, J. T., E. B. Kegley, J. G. Powell, P. A. Beck, B. L. Vander Ley, and J. F. Ridpath. 2012. Weaning management of newly received beef calves with or without continuous exposure to a persistently infected bovine viral diarrhea pen mate: effects on health, performance, bovine diarrhea virus titers, and peripheral blood leukocytes. J. Anim. Sci. 90:1972–1985. doi:10.2527/jas2011-4077 22648754 PMC7110029

[CIT0093] Richeson, J. T., and T. R. Falkner. 2020. Bovine respiratory disease vaccination: what is the effect of timing? Vet. Clin. North Am. Food Anim. Pract. 36:473–485. doi:10.1016/j.cvfa.2020.03.013 32451036

[CIT0094] Richeson, J. T., T. E. Lawrence, and B. J. White. 2018. Using advanced technologies to quantify beef cattle behavior. Transl. Anim. Sci. 2:223–229. doi:10.1093/tas/txy004 32704706 PMC7200524

[CIT0095] Richeson, J. T., K. L. Samuelson, and D. J. Tomczak. 2019. Beef species–ruminant nutrition cactus beef symposium: energy and roughage levels in cattle receiving diets and impacts on health, performance, and immune responses. J. Anim. Sci. 97:3596–3604. doi:10.1093/jas/skz159 31074787 PMC6667253

[CIT0096] Rivera, J. D., M. L. Galyean, and W. T. Nichols. 2005. Review: dietary roughage concentration and health of newly received cattle. Prof. Anim. Sci. 21:345–351. doi:10.15232/S1080-7446(15)31231-6

[CIT0097] Rivera, J. D., J. T. Johnson, and G. K. Blue. 2018. Effects of oral tilmicosin on health and performance in newly received beef heifers. Prof. Anim. Sci. 34:42–50. doi:10.15232/pas.2017-01639

[CIT0098] Rivera, J. D., J. T. Johnson, and M. D. Cravey. 2019. Effects of yeast and yeast cell wall on performance and health of newly received beef steers and heifers grazing bahiagrass pastures. Prof. Anim. Sci. 35:339–346. doi:10.15232/aas.2018-01804

[CIT0099] Roberts, S. L., N. D. May, C. L. Brauer, W. W. Gentry, C. P. Weiss, J. S. Jennings, and J. T. Richeson. 2016. Effect of injectable trace mineral administration on health, performance, and vaccine response of newly received feedlot cattle. Prof. Anim. Sci. 32:842–848. doi:10.15232/pas.2016-01543

[CIT0100] Ryan, A. W., E. B. Kegley, J. Hawley, J. G. Powell, J. A. Hornsby, J. L. Reynolds, and S. B. Laudert. 2015. Supplemental trace minerals (zinc, copper, and manganese) as sulfates, organic amino acid complexes, or hydroxy trace mineral sources for shipping-stressed calves. Prof. Anim. Sci. 31:333–341. doi:10.15232/pas.2014-01383 32288477 PMC7147669

[CIT0101] Samuelson, K. L., M. E. Hubbert, M. L. Galyean, and C. A. Löest. 2016. Nutritional recommendations of feedlot consulting nutritionists: The 2015 New Mexico State and Texas Tech University survey. J. Anim. Sci. 94:2648–2663. doi:10.2527/jas.2016-0282 27285940

[CIT0102] Schneider, C. J., B. L. Nuttelman, W. A. Griffin, D. B. Burken, D. R. Smith, T. J. Klopfenstein, and G. E. Erickson. 2013. Using RAMP® for receiving cattle compared to traditional receiving diets. 2013 Nebraska Beef Cattle Report. Lincoln (NE): University of Nebraska, pp 84–85.

[CIT0103] Schneider, C. J., B. L. Nuttelman, K. M. Rolfe, W. A. Griffin, D. R. Smith, T. J. Klopfenstein, and G. E. Erickson. 2012. Use of complete-feed diets RAMP™ and test starter for receiving cattle. 2012 Nebraska Beef Cattle Report. Lincoln (NE): University of Nebraska, pp 87–88.

[CIT0104] Schumacher, T. F., R. F. Cooke, A. P. Brandão, L. M. Schubach, O. A. de Sousa, D. W. Bohnert, and R. S. Marques. 2019. Effects of vaccination timing against respiratory pathogens on performance, antibody response, and health of feedlot cattle. J. Anim. Sci. 97:620–630. doi:10.1093/jas/sky466 30517650 PMC6358237

[CIT0105] Schwartzkopf-Genswein, K., J. Ahola, L. Edwards-Callaway, D. Hale, and J. Paterson. 2016. Symposium paper: transportation issues affecting cattle well-being and considerations for the future. Prof. Anim. Sci. 32:707–716. doi:10.15232/pas.2016-01517

[CIT0106] Sgoifo Rossi, C. A., R. Compiani, G. Baldi, M. Muraro, J. P. Marden, R. Rossi, G. Pastorelli, C. Corino, and V. Del’Orto. 2017. Organic selenium supplementation improves growth parameters, immune and antioxidant status of newly received beef cattle. J. Anim. Feed Sci. 26:100–108. doi:10.22358/jafs/70765/20

[CIT0107] Sharon, K., G. Duff, J. Dailey, J. Carroll, J. Hilmer, and B. Bothner. 2014. Effects of supplemental lysine on performance, antibody titer and rectal temperature in response to a modified-live viral vaccine in neonatal calves. Am. J. Anim. Vet. Sci. 9:122–127. doi:10.3844/ajavsp.2014.122.127

[CIT0108] Sharon, K. P., G. C. Duff, J. A. Paterson, J. W. Dailey, J. A. Carroll, and E. A. Marceau. 2013. Case study: effects of timing of a modified-live respiratory viral vaccination on performance, feed intake, antibody titer response, and febrile response of beef heifers. Prof. Anim. Sci. 29:307–312. doi:10.15232/S1080-7446(15)30237-0

[CIT0109] Sharman, E. D., J. J. Wagner, C. K. Larson, J. S. Schutz, N. E. Davis, and T. E. Engle. 2008. The effects of trace mineral source on performance and health of newly received steers and the impact of cobalt concentration on performance and lipid metabolism during the finishing phase. Prof. Anim. Sci. 24:430–438. doi:10.15232/S1080-7446(15)30885-8

[CIT0110] Smerchek, D. T., E. M. Buckhaus, K. D. Miller, and Z. K. Smith. 2020. Increasing hay inclusion in silage-based receiving diets and its effects on performance and energy utilization in newly weaned beef steers. Transl. Anim. Sci. 4:txaa026. doi:10.1093/tas/txaa026 32705024 PMC7201166

[CIT0111] Smith, Z. K., K. Karges, and A. Aguilar. 2020. Evaluation of an active live yeast (Levucell *Saccharomyces cerevisiae*, CNCM 1-1077) on receiving and backgrounding period growth performance and efficiency of dietary net energy utilization in low health risk beef steers. Transl. Anim. Sci. 4:1–7. doi:10.1093/tas/txaa127 32766530 PMC7398567

[CIT0112] Smock, T. M., K. L. Samuelson, J. E. Hergenreder, P. W. Rounds, and J. T. Richeson. 2020. Effects of *Bacillus subtilis* PB6 and/or chromium propionate supplementation on clinical health, growth performance, and carcass traits of high-risk cattle during the feedlot receiving and finishing periods. Transl. Anim. Sci. 4:txaa163. doi:10.1093/tas/txaa163 33134873 PMC7584392

[CIT0113] Snyder, E., B. Credille, R. Berghaus, and S. Giguѐre. 2017. Prevalence of multi drug antimicrobial resistance in *Mannheimia haemolytica* isolated from high-risk stocker cattle at arrival and two weeks after processing. J. Anim. Sci. 95:1124–1131. doi:10.2527/jas2016.1110 28380515

[CIT0114] Spore, T. J., S. P. Montgomery, E. C. Titgemeyer, G. A. Hanzlicek, C. I. Vahl, T. G. Nagaraja, K. T. Cavalli, W. R. Hollenbeck, R. A. Wahl, and D. A. Blasi. 2018. Effects of dietary energy level and intake of corn by-product-based diets on newly received growing cattle: antibody production, acute phase protein response, stress, and immunocompetency of healthy and morbid animals. J. Anim. Sci. 96:1474–1483. doi:10.1093/jas/sky035 29471465 PMC6140877

[CIT0115] Spore, T. J., S. P. Montgomery, E. C. Titgemeyer, G. A. Hanzlicek, C. I. Vahl, T. G. Nagaraja, K. T. Cavalli, W. R. Hollenbeck, R. A. Wahl, and D. A. Blasi. 2019. Effects of a high-energy programmed feeding protocol on nutrient digestibility, health, and performance of newly received growing beef cattle. Appl. Anim. Sci. 35:397–407. doi:10.15232/aas.2019-01853

[CIT0116] Tennant, T. C., S. E. Ives, L. B. Harper, D. G. Renter, and T. E. Lawrence. 2014. Comparison of tulathromycin and tilmicosin on the prevalence and severity of bovine respiratory disease in feedlot cattle in association with feedlot performance, carcass characteristics, and economic factors. J. Anim. Sci. 92:5203–5213. doi:10.2527/jas.2014-7814 25349362

[CIT0117] Theurer, M. E., J. T. Fox, L. K. Bryant, J. S. Nickell, and J. P. Hutcheson. 2018. Treatment efficacy of tildipirosin or tulathromycin for first treatment of naturally occurring bovine respiratory disease in a commercial feedlot. Bov. Pract. 52:154–159. doi:10.21423/bovine-vol52no2p154-159

[CIT0118] Theurer, M. E., M. D. Johnson, T. Fox, T. M. McCarty, R. M. McCollum, T. M. Jones, and D. O. Alkire. 2021. Bovine respiratory disease during the mid-portion of the feeding period: observations of frequency, timing, and population from the field. Appl. Anim. Sci. 37:52–58. doi:10.15232/aas.2020-02089

[CIT0119] Thrift, F. A., and T. A. Thrift. 2011. Review: update on preconditioning beef calves prior to sale by cow-calf producers. Prof Anim. Sci. 27:73–82. doi:10.15232/S1080-7446(15)30452-6

[CIT0120] Tomczak, D. J., K. L. Samuelson, J. S. Jennings, and J. T. Richeson. 2019. Oral hydration therapy with water affects health and performance of high-risk, newly received feedlot cattle. Appl. Anim. Sci. 35:30–38. doi:10.15232/aas.2018-01796 PMC6488335 30911760

[CIT0121] Urban-Chmiel, R., P. Hola, U. Lisiecka, A. Wernicki, A. Puchalski, M. Dec, and M. Wysocka. 2011. An evaluation of the effects of α-tocopherol and ascorbic acid in bovine respiratory disease complex occurring in feedlot calves after transport. Livest. Sci. 141:53–58. doi:10.1016/j.livsci.2011.05.003

[CIT0122] Van Donkersgoed, J., and J. K. Merrill. 2013. Efficacy of tilmicosin and tildipirosin for on-arrival treatment of bovine respiratory disease in fall-placed feedlot calves in western Canada. Bov. Pract. 47:146–151. doi:10.21423/bovine-vol47no2p146-151

[CIT0123] Van Engen, N. K., T. J. Engelken, C. G. Lockard, J. Lakritz, N. Cernicchiaro, B. K. Wilson, C. R. Krehbiel, and J. F. Coetzee. 2019. The effects of pretransportation or arrival meloxicam administration to calves entering the feedlot on morbidity, biomarkers, performance, and carcass characteristics. Transl. Anim. Sci. 3:620–632. doi:10.1093/tas/txz070 32704832 PMC7200945

[CIT0124] Vendramini, J. M. B., and J. D. Arthington. 2007. Case study: effects of supplemental yeast fermentation product on performance of early weaned calves on pasture and measures of stress and performance during a feedlot receiving period. Prof. Anim. Sci. 23:709–714. doi:10.15232/S1080-7446(15)31044-5

[CIT0125] Waggoner, J. W., C. A. Löest, J. L. Turner, C. P. Mathis, and D. M. Hallford. 2009. Effects of dietary protein and bacterial lipopolysaccharide infusion on nitrogen metabolism and hormonal responses of growing beef steers. J. Anim. Sci. 87:3656–3668. doi:10.2527/jas.2009-2011 19648488

[CIT0126] Wahrmund, J. L., D. B. Burken, B. K. Wilson, S. J. Terrill, D. L. Step, C. R. Krehbiel, S. M. Trost, and C. J. Richards. 2012. Case study: effect of truck compartment on ruminal temperature during transit and subsequent health and performance of newly weaned beef heifers. Prof. Anim. Sci. 28:670–677. doi:10.15232/S1080-7446(15)30427-7

[CIT0127] Weary, D. M., J. M. Huzzey, and M. A. von Keyserlingk. 2009. Board-invited review: using behavior to predict and identify ill health in animals. J. Anim. Sci. 87:770–777. doi:10.2527/jas.2008-1297 18952731

[CIT0128] White, B. J., and D. G. Renter. 2009. Bayesian estimation of the performance of using clinical observations and harvest lung lesions for diagnosing bovine respiratory disease in post-weaned beef calves. J. Vet. Diagn. Invest. 21:446–453. doi:10.1177/104063870902100405 19564492

[CIT0129] Whitney, T. R., G. C. Duff, J. K. Collins, D. W. Schafer, and D. M. Hallford. 2006. Effects of diet for early-weaned crossbred beef steers on metabolic profiles and febrile response to an infectious bovine herpesvirus-1 challenge. Livest. Sci. 101:1–9. doi:10.1016/j.livprodsci.2005.04.011

[CIT0130] Wiegand, J. B., R. F. Cooke, A. P. Brandão, K. M. Schubach, E. A. Colombo, C. L. Daigle, G. C. Duff, and V. N. Gouvêa. 2020. Impacts of commingling cattle from different sources on their physiological, health, and performance responses during feedlot receiving. Transl. Anim. Sci. 4:txaa204. doi:10.1093/tas/txaa204 33354658 PMC7743617

[CIT0131] Wilson, B. K., C. J. Richards, D. L. Step, and C. R. Krehbiel. 2017a. Best management practices for newly weaned calves for improved health and well-being. J. Anim. Sci. 95:2170–2182. doi:10.2527/jas.2016.1006 28727007

[CIT0132] Wilson, B. K., D. L. Step, C. L. Maxwell, C. A. Gifford, C. J. Richards, and C. R. Krehbiel. 2017b. Effect of bovine respiratory disease during the receiving period on steer finishing performance, efficiency, carcass characteristics, and lung scores. Prof. Anim. Sci. 33:24–36. doi:10.15232/pas.2016-01554 32288478 PMC7147665

[CIT0133] Wolfger, B., K. S. Schwartzkopf-Genswein, H. W. Barkema, E. A. Pajor, M. Levy, and K. Orsel. 2015. Feeding behavior as an early predictor of bovine respiratory disease in North American feedlot systems. J. Anim. Sci. 93:377–385. doi:10.2527/jas.2013-8030 25568380

[CIT0134] Woolsoncroft, M. A., M. E. Youngers, L. J. McPhillips, C. G. Lockard, C. L. Haviland, E. S. DeSocio, W. R. Ryan, C. J. Richards, and B. K. Wilson. 2018. Effects of exercise and roughage source on the health and performance of receiving beef calves. Prof. Anim. Sci. 34:183–191. doi:10.15232/pas.2017-01673

[CIT0135] Word, A. B., P. R. Broadway, N. C. Burdick Sanchez, S. L. Roberts, J. T. Richeson, Y. L. Liang, B. P. Holland, M. D. Cravey, J. R. Corley, M. A. Ballou, et al. 2019. Immune and metabolic responses of beef heifers supplemented with *Saccharomyces cerevisiae* to a combined viral-bacterial respiratory disease challenge. Transl. Anim. Sci. 3:135–148. doi:10.1093/tas/txy117 32704786 PMC7200475

[CIT0136] Word, A. B., G. B. Ellis, B. P. Holland, M. N. Streeter, and J. P. Hutcheson. 2021. Effects of antimicrobial metaphylaxis using no antimicrobial, tilmicosin, or tildipirosin and 2 different days on feed on the health and growth performance of lightweight beef steer calves originating from Mexico. Appl. Anim. Sci. 37:207–216. doi:10.15232/aas.2020-02117

[CIT0137] Word, A. B., T. A. Wickersham, L. A. Trubenbach, G. B. Mays, and J. E. Sawyer. 2020. Effects of metaphylaxis on production responses and total antimicrobial use in high-risk beef calves. Appl. Anim. Sci. 36:265–270. doi:10.15232/aas.2019-01914

[CIT0138] Young, T. R., F. N. B. Ribeiro, N. C. Burdick Sanchez, J. A. Carroll, M. A. Jennings, J. T. Cribbs, R. J. Rathmann, J. R. Corley, and B. J. Johnson. 2017. Yeast cell wall supplementation alters the performance and health of beef heifers during the receiving period. Prof. Anim. Sci. 33:166–175. doi:10.15232/pas.2016-01511

[CIT0139] Zeineldin, M., R. Barakat, A. Elolimy, A. Z. M. Salem, M. M. Y. Elghandour, and J. C. Monroy. 2018. Synergetic action between the rumen microbiota and bovine health. Microb. Pathog. 124:106–115. doi:10.1016/j.micpath.2018.08.038 30138752

